# Fluorescence Correlation Spectroscopy Reveals Survival Motor Neuron Oligomerization but No Active Transport in Motor Axons of a Zebrafish Model for Spinal Muscular Atrophy

**DOI:** 10.3389/fcell.2021.639904

**Published:** 2021-08-11

**Authors:** Angela Koh, Menachem Viktor Sarusie, Jürgen Ohmer, Utz Fischer, Christoph Winkler, Thorsten Wohland

**Affiliations:** ^1^Department of Biological Sciences, Centre for Bioimaging Sciences, National University of Singapore, Singapore, Singapore; ^2^Department of Biochemistry, Theodor-Boveri-Institut für Biowissenschaften (Biozentrum), University of Wuerzburg, Wuerzburg, Germany; ^3^Department of Chemistry, National University of Singapore, Singapore, Singapore

**Keywords:** fluorescence correlation spectroscopy, spinal muscular atrophy, survival motion neuron, zebrafish, motor axons, active transport, smn oligomerization

## Abstract

Spinal Muscular Atrophy (SMA) is a progressive neurodegenerative disease affecting lower motor neurons that is caused by a deficiency in ubiquitously expressed Survival Motor Neuron (SMN) protein. Two mutually exclusive hypotheses have been discussed to explain increased motor neuron vulnerability in SMA. Reduced SMN levels have been proposed to lead to defective snRNP assembly and aberrant splicing of transcripts that are essential for motor neuron maintenance. An alternative hypothesis proposes a motor neuron-specific function for SMN in axonal transport of mRNAs and/or RNPs. To address these possibilities, we used a novel *in vivo* approach with fluorescence correlation spectroscopy (FCS) in transgenic zebrafish embryos to assess the subcellular dynamics of Smn in motor neuron cell bodies and axons. Using fluorescently tagged Smn we show that it exists as two freely diffusing components, a monomeric, and a complex-bound, likely oligomeric, component. This oligomer hypothesis was supported by the disappearance of the complex-bound form for a truncated Smn variant that is deficient in oligomerization and a change in its dynamics under endogenous Smn deficient conditions. Surprisingly, our FCS measurements did not provide any evidence for an active transport of Smn in axons. Instead, our *in vivo* observations are consistent with previous findings that SMN acts as a chaperone for the assembly of snRNP and mRNP complexes.

## Introduction

Spinal Muscular Atrophy (SMA) is an autosomal recessive neurodegenerative disease characterized by the loss of alpha motor neurons in the anterior horn of the lower spinal cord. This loss leads to progressive wasting of proximal muscles, paralysis and eventually death. A majority of SMA patients (>95%) have a homozygous deletion of the causative gene, Survival Motor Neuron 1 (*SMN1*) (Lefebvre et al., 1995).

In humans, there are two copies of *SMN* on chromosome 5q13: The telomeric copy, *SMN1* and a more centromeric inverted copy, *SMN2*. The latter produces only ∼10% of full-length transcripts (*FL-SMN2*) and 90% of alternatively spliced transcripts lacking exon 7 (*SMN2Δ7*). Exon 7 skipped transcripts encode an unstable, truncated protein that shows reduced self-oligomerization, and is rapidly degraded (Lorson et al., 1998; Pellizzoni et al., 1999). Hence, *SMN2* cannot fully compensate for the homozygous loss of *SMN1*. The severity of SMA phenotypes is inversely correlated to *SMN2* copy number (Feldkotter et al., 2002), thus this suggests that it is dependent on levels of functional SMN. However, it remains unclear why the reduction of ubiquitously expressed SMN primarily leads to the degeneration of motor neurons.

The first of two main hypotheses to explain this selective vulnerability of motor neurons (Burghes and Beattie, 2009) is based on SMN’s established role in the biogenesis of small nuclear ribonucleoproteins (snRNPs) (Pellizzoni et al., 2002). snRNPs are RNA-protein complexes, which are composed of snRNA, Sm proteins (B/B’, D1, D2, D3, E, F, and G) and specific proteins that are unique for each snRNP. In the first of multiple steps in this biogenesis, the SMN complex acts as a catalyst to assemble Sm proteins into a heptameric ring on the snRNA (Chari et al., 2008). It is proposed that reduced SMN levels affect proper snRNP assembly, leading to aberrant splicing of transcripts essential for motor neurons (Winkler et al., 2005; Chari et al., 2009; See et al., 2014). A second hypothesis suggests an additional role of SMN exclusively in motor neurons that is independent of snRNP assembly. Consistent with this, SMN protein was detected in dendrites (Bechade et al., 1999), neurites (Fan and Simard, 2002) and axons of cultured motor neurons (Pagliardini et al., 2000; Rossoll et al., 2002). A gradient of SMN localization from nuclei to axons was shown in spinal cords of postmortem fetuses (Giavazzi et al., 2006). In addition, imaging of cultured chick forebrain neurons revealed rapid and bidirectional movement of fluorescently tagged SMN granules along axons (Zhang et al., 2003). SMN was also found to be trafficking in a retro- and anterograde fashion in axons of primary mouse motor neurons (Fallini et al., 2010). SMN interacts with many proteins that have been implicated in axonal β-*actin* mRNA transport and regulation of actin dynamics (Rossoll et al., 2002; Glinka et al., 2010). Accordingly, a SMN-mRNA granule hypothesis has been proposed (Rossoll and Bassell, 2009), in which SMN interacts with RNA binding proteins to form RNP complexes responsible for the localization of β-*actin* mRNA and other transcripts to axons and growth cones.

Survival motor neuron has been found to be involved in other processes. It was shown to function as a molecular chaperone for the assembly of IMP1 protein with β*-actin* mRNA into messenger ribonucleoproteins (mRNPs) (Donlin-Asp et al., 2017). In addition, the RNA binding protein FUS, which is mutated in Amyotrophic lateral sclerosis (ALS), associates with the SMN complex via direct interaction with SMN, suggesting a common biochemical pathway between ALS and SMN (Yamazaki et al., 2012). SMN was also found to be involved in the regulation of local axonal translation via the miR-183/mTOR pathway (Kye et al., 2014). More recently, it has been shown that SMN associates with ribosomes and has a role in regulating the translation of a specific subset of mRNAs that are related to SMA (Lauria et al., 2020).

In this study, we investigated Smn dynamics by Fluorescence Correlation Spectroscopy (FCS) in cell culture and *in vivo* in zebrafish embryos. FCS is a technique with single-molecule sensitivity and is thus preferable over conventional life imaging of cultured neurons *in vitro*, used previously to suggest axonal transport of Smn. The use of this technique bridges the gap that cannot be addressed by biochemical analyses, that is, to observe movement of molecular particles *in vivo.* FCS has been used to show interactions and to confirm biochemical data obtained *in vitro* or *in vivo* (Mistri et al., 2015). Hence this provides us with a novel technique to address the question of Smn dynamics *in vivo*.

For this, Smn was fluorescently tagged with mCherry or eGFP and expressed under control of the *mnx1*/*hb9* promoter in motor neurons of zebrafish. FCS measurements showed that mCherry-Smn and eGFP-Smn diffused in the cytoplasm of somata and axons as freely diffusing particles, and as slow-moving complex bound particles. The latter became faster in axons and was not detected in the cell body when a truncated, oligomerization deficient eGFP-SmnΔex6,7 was analyzed. This demonstrated that the slow-moving component represents the oligomeric form of Smn. Importantly, in contrast to earlier cell culture findings, no active transport of Smn in axons was detected by FCS measurements.

## Materials and Methods

### Zebrafish Care and Maintenance

Zebrafish were bred and maintained in the fish facility of the Department of Biological Sciences, National University of Singapore (NUS) according to approved protocols (protocol no BR15-0119; R13-574; R17-1522) under the Institute of Animal Care and Use Committee (IACUC) guidelines. Adult fish were raised under a 14 h light/10 h dark cycle, with regular monitoring of water quality, temperature, pH and salinity. The transgenic line, *hb9*:mCherry-linker-Smn, expresses a mCherry-Smn fusion protein in motor neurons of zebrafish and was generated previously (Spiró et al., 2016).

### Cloning of Plasmids

#### *hb9*:eGFP-Linker-Smn

To study Smn dynamics, *hb9*:eGFP-linker-Smn was derived from *hb9*:eGFP (Spiró et al., 2016) and *hb9*:mCherry-linker-Smn (Spiró et al., 2016), both containing *I-SceI* sites for efficient transgenesis. First, the stop codon was removed from eGFP by PCR amplification using a forward primer containing a *BamHI* site and Kozak sequence, and a reverse primer recognizing the 3′ end of eGFP, containing an *EcoRI* site but lacking the stop codon. The resultant eGFPΔTAA amplicon and *hb9*:eGFP plasmid were sequentially digested with *BamHI* and *EcoRI*, and gel purified. Next, eGFPΔTAA was ligated into the *hb9* vector backbone to generate *hb9*:eGFPΔTAA. The linker-Smn fragment was obtained by digesting *hb9*:mCherry-linker-Smn with *EcoRI*. Concurrently, *hb9*:eGFPΔTAA was digested with *EcoRI*. Finally, the linker-Smn fragment was ligated into *hb9*:eGFPΔTAA to obtain *hb9*:eGFP-linker-Smn.

#### *hb9*:eGFP-Linker-SmnΔex6,7

To obtain *hb9*:eGFP-linker-SmnΔex6,7, which lacks *smn* exons 6 and 7, a reverse primer targeting the end of exon 5, and containing a stop codon and an *EcoRI* site was designed. The zebrafish cDNA sequence (ENSDARG00000014569.7) obtained from ensembl.org (zebrafish genome GRCv9) was used for designing the primer. The linker-SmnΔex6,7 fragment was amplified using the newly designed reverse primer and a forward primer containing *EcoRI* and a (SGGG)_3_ linker. The backbone *hb9*:eGFP-linker-Smn was digested with *EcoRI* and gel purified to separate the linker-Smn insert from the backbone *hb9*:eGFPΔTAA, which was then dephosphorylated and purified. The linker-SmnΔex67 amplicon was digested with *EcoRI* and inserted into *hb9*:eGFPΔTAA to obtain *hb9*:eGFP-linker-SmnΔex67.

### Cell Culture and Transfection

SH-SY5Y neuroblastoma cells were obtained from ATCC (Manassas, VA, United States) and cultivated in DMEM (Dulbecco’s Modified Eagle Medium; HyClone, GE Healthcare Life Sciences, South Logan, UT, United States) supplemented with 10% FBS (fetal bovine serum; HyClone, GE Healthcare Life Sciences, South Logan, UT, United States) and 1% PS (penicillin and streptomycin, PAA, Austria), at 37°C in a 5% (v/v) CO_2_ humidified environment. Plasmids encoding mCherry, mCherry-Smn, and Smn were transfected into SH-SY5Y cells by electroporation using the Neon^TM^ Transfection System from Life Technologies (Grand Island, NY, United States). SH-SY5Y cells, at ∼80–90% confluency, cultured in a 25 cm^2^ culture flask were washed twice with 1× PBS and trypsinized by incubation with 0.5 mL of 1× Trypsin solution for 1 min at 37°C. The cells were checked under a microscope for full dislodge and trypsinization process was stopped by re-suspending cells with 3.5 mL of culture medium. The cell suspension was transferred to a 15 mL Falcon tube and then pelleted by centrifugation at 1,000 rpm for 3 min. Next, the cell pellet was re-suspended with 10 μL of R buffer, mixed with the appropriate amount of plasmids (125–250 ng) and drawn into a 10 μL Neon^TM^ transfection tip. Electroporation was carried out using conditions according to Neon^TM^ Protocol (Pulse voltage = 1,200 V, Pulse width = 20 ms, Pulse number = 3) for the SH-SY5Y cell line in a transfection tube containing 3 mL of E buffer.

After transfection, cells were plated onto a 35 mm glass bottom dish (MatTek Corporation) and grown in culture medium (DMEM and 10% FBS) at 37°C in a 5% (v/v) CO_2_ humidified environment. Confocal Fluorescence Correlation Spectroscopy (FCS) measurements were conducted 48–60 h post-transfection in 1× colorless DMEM. Transfected SH-SY5Y cells were washed twice with 1× PBS and filled with 1× colorless DMEM before measurements.

### Recombinant Protein Expression and Oligomerization Assays

GST-*h*SMN and MBP-*zf*SMN fusion proteins were expressed in *Escherichia coli* BL21(DE3) cells as described previously (Veepaschit et al., 2021). Both the fusion proteins also contained a His tag for purification on NiNTA column. In brief, cells were cultured in SB media at 37°C and induced with 1 mM IPTG (Isopropyl-β-D-thiogalactopyranoside) at an OD_600_ of 0.8. Bacteria cells were harvested by centrifugation after 18 h at 15°C and resuspended in buffer A [25 mM HEPES-NaOH (pH 7.0), 10 mM imidazole, 250 mM NaCl and 5 mM β-mercaptoethanol] containing protease inhibitors and lysed by sonication. Following ultracentrifugation at 185,000 *g* for 1 h at 4°C, the cleared lysate was incubated with pre-equilibrated NiNTA (Qiagen) for 2 h at 4°C. After extensive washing with buffer A, proteins were eluted with buffer B [25 mM HEPES-NaOH (pH 7.0), 350 mM imidazole, 250 mM NaCl and 5 mM β -mercaptoethanol]. The oligomerization of SMN proteins was investigated by size exclusion chromatography (Superdex^TM^ 200 Increase 10/300 GL, GE Healthcare) as described previously (Neuenkirchen et al., 2015).

### Confocal Fluorescence Correlation Spectroscopy (FCS) Instrumentation

Confocal FCS experiments were performed using an Olympus FV1200 confocal laser scanning microscope (Olympus, Tokyo, Japan). The green laser beam from a 543 nm helium-neon laser (Melles Griot, Singapore) or blue laser beam from 488 nm Argon-ion multi-line laser (Melles Griot, Singapore) passed the confocal scanning unit, with an intrinsic 3× magnification reflected by a dichroic mirror (405/488/543/635BP, Omega, VT) and focused into the sample by a water immersion objective (60×, NA 1.2; Olympus, Tokyo, Japan). The laser power before the objective was measured to be 6.5 μW. The fluorescence signal from the sample was passed through the same objective, de-scanned and finally sent through a 120 μm pinhole in the image plane to block the out-of-focus light. The light was then detected by an avalanche photodiode detector (APD) (SPCM-AQR-14; Perkin Elmer, Canada) after passing through a band pass emission filter 600/50 (Omega, Brattleboro, VT, United States) or 513/17 (Semrock, Rochester, NY, United States). The temporal signal from the APD was processed offline by a software correlator (SymPhoTime 64, PicoQuant TimeHarp 260) to generate an autocorrelation function (ACF).

### Data Acquisition and Fitting for SH-SY5Y Cells

On each cell, measurements were taken at three random spots in both nucleus and cytoplasm for 30–60 s. For each sample, measurements were taken in at least three cells. The ACFs were fitted using the SymPhoTime 64 software (PicoQuant) with a 3 Dimension 1-Particle (3D-1P) and triplet model (Equation 1a) for mCherry and a 3 Dimension 2-Particle (3D-2P) and triplet model (Equation 1b) for mCherry-Smn.

(1a) $$G\,\left(\tau \right)=\frac{1}{N}\,\left(1+\left(\frac{F_{\text{trip}}}{1+F_{\text{trip}}}\right)\,{\text{e}}^{-\tau /\tau_{\text{trip}}}\right)\,{\left(1+\frac{\tau }{\tau_{\text{D}}}\right)}^{-1}{\left(1+\frac{\tau }{K^{2}\,\tau_{\text{D}}}\right)}^{-1/2}+G_{\infty }$$

(1b) $$G\,\left(\tau \right)=\frac{1}{N}\left(1+\left(\frac{F_{\text{trip}}}{1+F_{\text{trip}}}\right)e^{-\tau /\tau_{\text{trip}}}\right)\{\left(1-F_{2}\right){\left(1+\frac{\tau }{\tau_{D_{1}}}\right)}^{-1}{\left(1+\frac{\tau }{K^{2}\,\tau_{D_{1}}}\right)}^{-1/2}+F_{2}{\left(1+\frac{\tau }{\tau_{D_{2}}}\right)}^{-1}{\left(1+\frac{\tau }{K^{2}\,\tau_{D_{2}}}\right)}^{-1/2}\}+G_{\infty }$$

*G*(*τ*) is the autocorrelation function (ACF) as a function of lag time (*τ*). *N* is the number of particles in the confocal observation volume; *τ*_*trip*_ describes the characteristic relaxation time of photophysical processes, including triplet transitions, with *F*_*trip*_ the fraction of particles undergoing these processes. Although there can be multiple photophysical processes, in general it is sufficient to describe them with one model. The average time of the *ith* species to pass through the observation volume is given by *τ*_*Di*_ and its mole fraction by *F*_*i*_. Note that *τ*_*trip*_ is typically much smaller than *τ*_*Di*_, and the photophysical and diffusive processes can be easily separated. We will therefore not discuss the photophysics in the rest of the manuscript and concentrate only on diffusive processes. *K* is the structure factor of the confocal volume defined as the ratio of the axial (*z*_0_) and radial (*w*_0_) distances where the intensity drops to 1/*e*^2^ of the maximum value. Finally, *G*_∞_ is the convergence value for the autocorrelation function at long lag times, which is expected to be 1, and in our measurements deviates not more than 3% from 1.

The diffusion coefficient (*D*) was determined from the Equation 2 where the *w*_0_ was obtained from the calibration measurement with Atto 565 in solution assuming its diffusion coefficient to be similar to that of Atto 488 which is 400 μm^2^/s (Kapusta, 2010).

(2) $$\tau_{D}=\frac{\omega_{0}^{2}}{4\,D}$$

Unless stated otherwise, we report all diffusion coefficient values as mean ± standard error of the mean (SEM) and statistical analyses were done using unpaired Student’s *t*-test.

### Zebrafish Microinjection and Sample Mounting

For transient expression of fluorescent proteins, zebrafish embryos were injected at the one-cell stage with plasmids together with *I-SceI* meganuclease according to established protocols (Rembold et al., 2006). For knockdown of endogenous Smn, Morpholino (MO), CGACATCTTCTGCACCATTGGC, obtained from GeneTools, United States, was injected at a concentration of 3.125 mg/ml into one-cell stage embryos, as described previously (Winkler et al., 2005). For injection of *mCherry-smn* mRNA, pCS2-mCherry-linker-smn was linearized with NotI and *in vitro* transcription of the capped mRNA was carried out using the mMessage mMachine Sp6 kit (Ambion). The transcribed mRNA was cleaned up by precipitation with 1 volume 3 M sodium acetate (pH5.2) in 1 volume isopropanol at −80°C. The resulting mRNA was injected at 100 ng/μL together with the *smn* MO.

Microinjections were carried out with a Femtojet Mircoinjector (Eppendorf) using glass capillaries (Harvard Apparatus), which had been pulled into sharp tips using a needle puller (Narishige, Japan).

Zebrafish embryos at 2 days post fertilization (2 dpf) were dechorionated using 27G × ½” needles (Terumo) and anesthetized for 15–30 min in 0.05% w/v tricaine (ethyl m-aminobenzoate, Sigma) in 1× Danieau’s medium. Embryos were mounted onto No. 1.0 glass bottom petri dishes (MatTek Corporation) with 1.2% low melting agarose (Bio-Rad Laboratories). To obtain lateral views, embryos were orientated using a microloader tip (Eppendorf) to lie as close to the glass slide as possible. Once the agarose had solidified, measurements on the confocal microscope were carried out.

### Data Acquisition and Fitting for Zebrafish Embryos

For each zebrafish embryo, three primary motor neurons expressing the fluorescent protein were randomly selected for measurements. In each motor neuron, measurements were taken for 30–60 s at two to three randomly chosen spots in the cytoplasm of the cell body and along the axon. For each sample, measurements were taken in at least three fish. The ACFs were fitted using the SymPhoTime 64 software (PicoQuant). The ACFs for mCherry were fitted starting from a lag time of 0.001 ms to a 3 Dimension 1-Particle (3D-1P) triplet model (Equation 1a). The triplet parameter does not only identify triplets but also any photophysical processes. For eGFP, eGFP-SmnΔex67, and mCherry-Smn analysis under knock-down conditions in the cell body, the ACFs were fitted starting from a lag time of 0.01 ms to a 3 Dimension 1-Particle (3D-1P) model (Equation 3a).

(3a) $$G\,\left(\tau \right)=\frac{1}{N}\,{\left(1+\frac{\tau }{\tau_{\text{D}}}\right)}^{-1}\,{\left(1+\frac{\tau }{K^{2}\,\tau_{\text{D}}}\right)}^{-1/2}+G_{\infty }$$

For mCherry-Smn, eGFP-Smn, eGFP-SmnΔex67, and mCherry-Smn analysis under MO condition in axons, the ACFs were fitted starting from lag time of 0.01 ms to a 3 Dimension 2-Particle (3D-2P) model (Equation 3b).

(3b) $$G\left(\tau \right)=\frac{1}{N}\{\left(1-F_{2}\right){\left(1+\frac{\tau }{\tau_{{\text{D}}_{1}}}\right)}^{-1}{\left(1+\frac{\tau }{K^{2}\,\tau_{{\text{D}}_{1}}}\right)}^{-1/2}+F_{2}{\left(1+\frac{\tau }{\tau_{{\text{D}}_{2}}}\right)}^{-1}{\left(1+\frac{\tau }{K^{2}\,\tau_{{\text{D}}_{2}}}\right)}^{-1/2}\}+G{}_{\infty }$$

The parameters in Equation 3a and 3b have the same definition as for Equation 1 and 2. The complex structure of the zebrafish embryo could have affected the actual shape and size of the observation volume during measurements, and absolute diffusion coefficients might have shifted from values seen in solution. However, our data on diffusion coefficients of fluorescent proteins in zebrafish are very comparable to cell measurements (see Supplementary Tables 1, 2), indicating that any influence of zebrafish tissue on the FCS measurements was limited. *F*_2_ is the mole fraction of the second component. However, to determine this correctly one needs to know the absolute brightness of the particles measured, as the contribution of a particle to the ACF depends on the square of its brightness. As the second components in this study are unknown, *F*_2_ here only reports on changes in either fraction or brightness. Unless stated otherwise, we report all diffusion coefficient values as mean ± standard error of the mean (SEM). Statistical analyses were done using unpaired Student’s *t*-test.

### Data Fitting for Blood Flow in Zebrafish

In each fish, measurements were taken for 60 s in the blood vessel near the heart region flowing into the yolk region. Autofluorescence of the blood were recorded in the intensity trace and translated into ACFs. The ACFs were fitted from a lagtime of 0.01 ms in software IgorPro 8.0 to a diffusion and flow model (Equation 4).

(4) $$G\,\left(\tau \right)=\frac{1}{N}\times {\left(1+\frac{\tau }{\tau_{\text{D}}}\right)}^{-1}\,{\left(1+\frac{\tau }{K^{2}\,\tau_{\text{D}}}\right)}^{-1/2}\times e\,x\,p\,\left[\frac{-{\left(\tau /\tau_{\text{f}}\right)}^{2}}{1+\left(\tau /\tau_{\text{D}}\right)}\right]+G_{\infty }$$

The parameters in Equation 4 have the same definition as for Equation 1 and 2. *τ*_*f*_ is the lag time for the flow process.

### Zebrafish Sample Fixation and Znp-1 Immunostaining

Larvae at 31 h post fertilization (hpf) were dechorionated using forceps or needles before fixation. 30–50 larvae per sample were transferred to a glass vial and medium was removed as much as possible, followed by the addition of 1 mL of 4% PFA/PBS. The samples were fixed for 4 h at room temperature (RT) on an orbital shaker (Grant Bio) or overnight at 4°C. The following day, the fixed samples were washed with 1× PBST for 5 min three times on a shaker at RT. For long-term storage at −20°C, they were washed in 100% methanol for 5 min on a shaker at RT and then replaced with fresh 100% methanol.

The PFA fixed embryos, which were kept in MeOH at −20°C, were rehydrated in 50% MeOH/H_2_O for 5 min, in Milli-Q water for 5 min and again in Milli-Q water for 1 h at RT on an orbital shaker (Grant Bio). They were blocked for 1 h in PBDT solution (1% DMSO, 1% BSA, 0.5% Triton × 100, 2.5% sheep serum, 1× PBS, 0.5% Tween-20). Samples were then incubated with a primary antibody directed against α-synaptotagmin2 (Znp1 monoclonal antibody, 1:100, ZIRC, United States) diluted in PBDT at 4°C overnight.

Next, the samples were washed in 0.1% TritonX in PBST at RT for 4 × 1 h each, before incubation with the secondary antibody, Biotin (1:1000; Vectastain ABC Elite-kit, Vector Labs, United States) overnight. Antibody detection for biotin-labeled samples was carried out prior to imaging in accordance with the Vectastain ABC Elite-kit protocol using DAB as substrate. For short-term storage, samples were kept in 1× PBST at 4°C.

### Image Acquisition for Znp-1 Immunostained Larvae

Znp-1 immunostained larvae were washed in 1× PBST, followed by incubation in 70% glycerol (AppliChem) for at least 1 h or overnight before mounting. Individual samples were dissected to separate the head from the trunk. The trunk was mounted on a microscope slide (Continental Lab Products) using 100% glycerol. The images were taken using a compound microscope (Nikon Eclipse 90i) in the Epifluorescence mode with a 20× objective. The NIS element basic software (Nikon) was used for image acquisition and analysis.

## Results

### mCherry-Smn Displays 2-Particle Diffusional Dynamics in SH-SY5Y Neuroblastoma Cells

**FIGURE 1:**
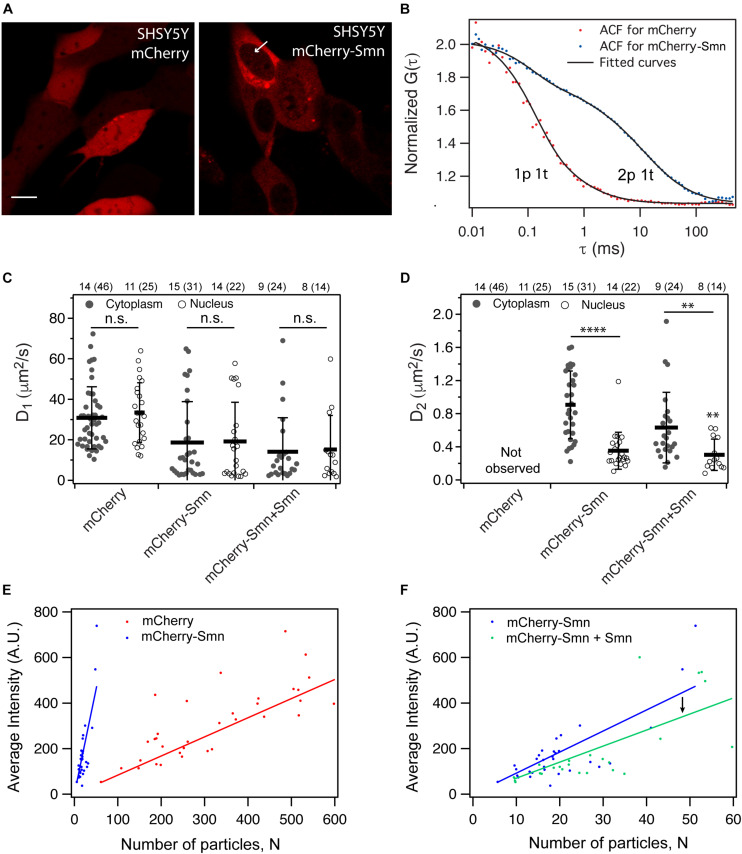
Expression and dynamics of mCherry and mCherry-Smn in SH-SY5Y cell culture. **(A)** Expression of mCherry and mCherry-Smn in SH-SY5Y cells 2 days after transfection. Scale bar = 10 μm. **(B)** Representative autocorrelation function (ACF) and fitted ACFs of mCherry and mCherry-Smn in the cytoplasm. ACF for mCherry is fitted to 3D 1p1t while ACF for mCherry-Smn is fitted to 3D 2p1t diffusion model. **(C)** Mean diffusion coefficients, D_1_ (μm^2^/s) in cytoplasm and nucleus for mCherry, mCherry-Smn and mCherry-Smn: Smn (mCherry-Smn co-transfected with untagged Smn). **(D)** Mean diffusion coefficients, D_2_ (μm^2^/s) in cytoplasm and nucleus. Error bars represent the SD. (^∗∗^*P* < 0.01, ^****^P < 0.0001). **(E)** Brightness analysis of mCherry and mCherry-Smn in cytoplasm by plotting average intensity vs. number of particles. mCherry-Smn particles are brighter than mCherry. **(F)** Brightness analysis of mCherry-Smn vs. mCherry-Smn with untagged Smn in the cytoplasm. With presence of untagged Smn, brightness of mCherry-Smn goes down. The numbers above the graphs indicate the number of cells (number of points) the measurements were taken in.

In a first step, we aimed at gaining a preliminary understanding of cellular Smn dynamics by performing FCS measurements on cultivated SH-SY5Y neuroblastoma cells. For this purpose, we used transiently expressed mCherry as a control molecule for cytosolic diffusion and mCherry-Smn as probe. We had previously shown that *hb9*:mCherry-Smn rescues motor neuron defects in *etvb5b* knock-down embryos, showing that mCherry-Smn is functional (Spiró et al., 2016). SH-SY5Y neuroblastoma cells were chosen as they are commonly used as *in vitro* system for neuron related studies, as a simple cell control. As expected, mCherry (∼27 kDa) was homogenously expressed throughout nucleus and cytoplasm in SH-SY5Y cells (Figure 1A), consistent with earlier reports that the size limit of proteins that diffuse through the nuclear pore is 90–110 kDa (Wang and Brattain, 2007). In contrast, mCherry-Smn was much less abundant in the nucleus but expressed strongly in the cytoplasm (Figure 1A). The size of mCherry-Smn is ∼60 kDa and thus should freely diffuse into the nucleus. Instead, we observed distinct spots of concentrated mCherry-Smn in the nucleus (white arrow), consistent with endogenous expression of SMN, suggesting that these could be structures known as gems where SMN is highly enriched in and commonly observed to co-localize with the Cajal bodies (Liu and Dreyfuss, 1996). It was shown that SMN is transported into the nucleus as a complex together with the assembled snRNPs using importers such as snurportin and importin β (Narayanan et al., 2002). This transport process is likely to be regulated, thus resulting in a relatively weak accumulation of mCherry-Smn within the nucleus.

Autocorrelation functions (ACFs) from confocal-FCS measurements were acquired and fitted to 3-dimensional (3D) diffusional models (Figure 1B). Figures 1C,D show average diffusion coefficients for mCherry, mCherry-Smn as well as mCherry-Smn in the presence of untagged Smn (mCherry-Smn + Smn), in the cytoplasm and nucleus. All diffusion coefficients are tabulated in Supplementary Table 1.

The ACFs of mCherry fitted well to a 3D 1-particle 1-triplet diffusion model (Figure 1B). Diffusion coefficients were similar at 30.8 ± 2.3 μm^2^/s in the cytoplasm and 33.4 ± 2.9 μm^2^/s in the nucleus (Figure 1C). In contrast, the ACFs for mCherry-Smn in both cytoplasm and nucleus fitted well only to 3D 2-particle 1-triplet diffusion models. The first component diffuses at 18.7 ± 3.6 μm^2^/s in the cytoplasm and at a similar rate of 19.2 ± 4.2 μm^2^/s in the nucleus. However, the second component diffuses at different rates in cytoplasm (0.91 ± 0.07 μm^2^/s) and nucleus (0.35 ± 0.05 μm^2^/s) (Figure 1D). From this point on, the first component will be referred to as ‘fast component’ (*D_1*) while the second component is the ‘slow component’ (*D_2*).

The diffusion coefficient of the fast component of mCherry-Smn was in the same order of magnitude as that of mCherry, albeit lower, likely because mCherry-Smn (∼60 kDa) is larger than mCherry (∼27 kDa) and because the two proteins likely have very different shapes. The slow component is slower than the fast component by two orders of magnitude.

To confirm whether the slow mCherry-Smn component could represent oligomers, we compared brightness of mCherry-Smn and mCherry particles, assuming that mCherry molecules are monomeric. Average intensities were plotted against the number of particles, *N*, a parameter obtained from the fitting results (Figure 1E). Hence, the slope of the curve reflects the brightness of particles. Molecular brightness is a measure of intensity per mobile particle and thus is normalized to the expression level. Therefore, while intensity may reflect expression levels of the particles, molecular brightness does not. A higher molecular brightness indicates a larger number of fluorescent molecules bound to the mobile particle.

The fitted curve for mCherry-Smn was much steeper than for mCherry, indicating that mCherry-Smn particles are brighter than mCherry molecules and thus suggesting that mCherry-Smn particles reflect oligomers. We then examined the dynamics of mCherry-Smn in the presence of an excess of untagged Smn by transfecting SH-SY5Y cells with equal amounts of 125 ng mCherry-Smn and 125 ng untagged Smn plasmids. The untagged Smn competes for binding to complexes or oligomers with mCherry-Smn. We assumed that if mCherry-Smn were replaced by untagged Smn in an oligomer or complex, the particle would decrease in brightness. Consistent with this, a downward shift in the fitted line was observed when untagged-Smn was present (Figure 1F). This supports the hypothesis of a competitive binding between mCherry-Smn and Smn and that mCherry-Smn oligomers are present in the slow particles.

To further support the oligomerization of mCherry-Smn, and to verify that zebrafish Smn can indeed function and interact with human SMN in SH-SY5Y cells, *in vitro* interaction assays between purified protein constructs were carried out. We showed that zebrafish Smn (His_6_-MBP-*zf*SMN*wt*) co-eluted with human SMN (GST-*h*SMN (252-284)) on size exclusion chromatography (Supplementary Figures 1C,C′). The human-zebrafish hybrid complex (Supplementary Figures 1C,C′) displays very similar oligomerization properties as the human-SMN only control (Supplementary Figures 1A,A′) and zebrafish-Smn only control (Supplementary Figures 1B,B′), which is in line with a recent report on SMN oligomerization (Veepaschit et al., 2021). We conclude that zebrafish Smn does interact and form oligomers with human SMN.

### Smn Dynamics Is Similar in Cell Bodies and Axons of Zebrafish Motor Neurons

**FIGURE 2:**
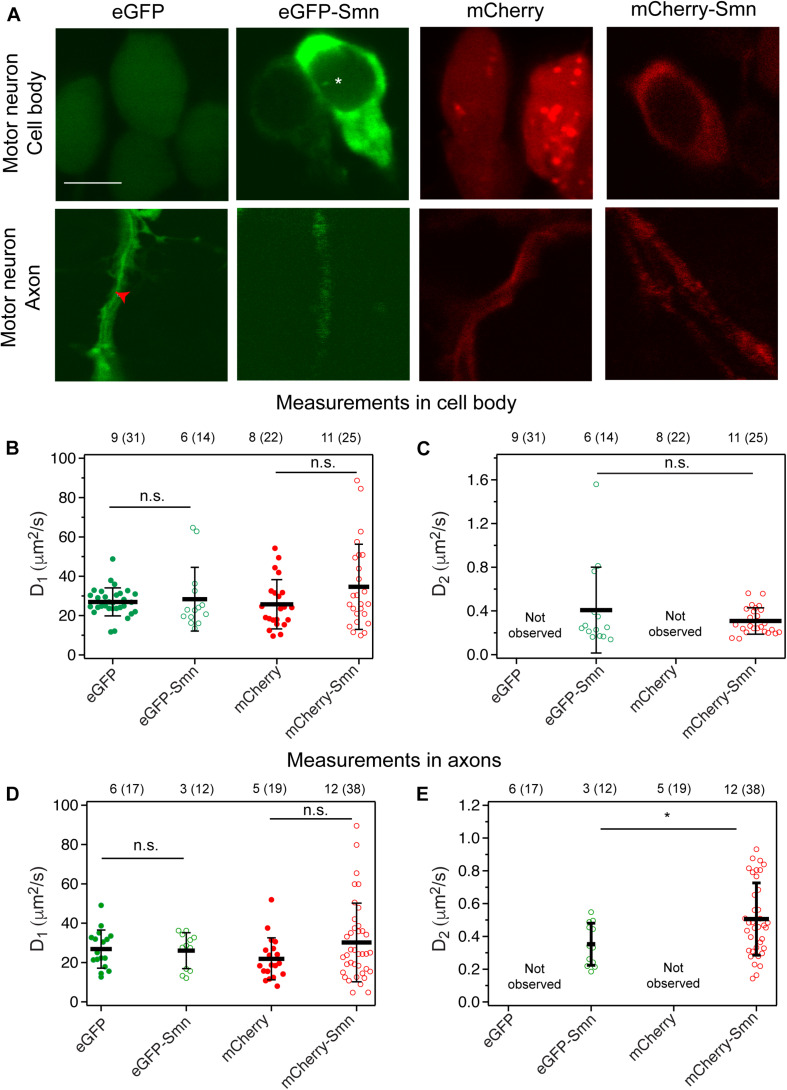
Expression and diffusion coefficients of FPs and FP-tagged Smn in zebrafish motor neurons. **(A)** Expression of eGFP, eGFP-Smn, mCherry, and mCherry-Smn in cell body and axon of motor neurons at 2 dpf. Images of cell body and axon are taken from different motor neurons. Scale bar = 5 μm. Red arrow indicates representative position where FCS measurement was taken in axon. **(B)** Average diffusion coefficients, D_1_ (μm^2^/s) of eGFP, eGFP-Smn, mCherry, mCherry-Smn in cell body. **(C)** Average diffusion coefficients, D_2_ (μm^2^/s) of eGFP-Smn, mCherry-Smn in cell body. **(D)** Average diffusion coefficients, D_1_ (μm^2^/s) of eGFP, eGFP-Smn, mCherry, mCherry-Smn in axons. **(E)** Average diffusion coefficients, D_2_ (μm^2^/s) of eGFP-Smn, mCherry-Smn in axons. Error bars represent the SD. (^*n.s.*^*P* > 0.05, *P < 0.05). The numbers above the graph indicate the numbers of fish (number of points) the measurements were taken in.

We next carried out FCS measurements of Smn tagged to mCherry or eGFP in zebrafish embryos *in vivo* at 2 dpf. The measurements were taken at 2 dpf as the motor axons are developed and the first wave of neuromuscular junctions has formed by this time point. Both fluorescent proteins (FPs) were used to verify that the dynamics of Smn was not affected by using different fluorophores. Transgenic *hb9*:mCherry and *hb9*:eGFP lines, which expressed FPs specifically in motor neurons, were used as controls. mCherry-Smn was stably expressed in the *hb9*:mCherry-Smn transgenic line, while eGFP-Smn was transiently expressed upon injecting the plasmid into one-cell stage embryos. Bright aggregates, above the level for the complexes in the previous section, were observed for mCherry 70% of the time, but not for eGFP, in motor neurons (Figure 2A), and these aggregates were avoided during FCS measurements. Similar to observations in cell culture, eGFP-Smn and mCherry-Smn were expressed more strongly in the cytoplasm than the nucleus, while both eGFP and mCherry were expressed homogeneously throughout both compartments (Figure 2A). We also observed regions with higher fluorescence intensity (asterisks) in the nucleus indicative of Cajal bodies where Smn is known to localize. Hence, the expression of the fluorescently tagged Smn in motor neurons is consistent with previous findings in cultured cells (Liu and Dreyfuss, 1996).

Next, FCS measurements were carried out at randomly selected locations in cell bodies and along axons. As expected, mCherry-Smn and eGFP-Smn were detected in motor axons consistent with earlier reports on endogenous SMN in axons of cultured motor neurons (Pagliardini et al., 2000; Rossoll et al., 2002) and transgenic zebrafish expressing human SMN (Hao et al., 2015). In both compartments, ACFs obtained from mCherry and eGFP measurements fitted well to a 3D 1-particle diffusion model, while ACFs for mCherry-Smn and eGFP-Smn could only be fitted to a 2-particle 3D diffusion model. Thus, similar results were obtained in zebrafish motor neuronal cell bodies and axons *in vivo* when compared to observations in SH-SY5Y cells.

The diffusion coefficients D_1_ of the fast components of eGFP-Smn and mCherry-Smn were in the same order of magnitude as eGFP and mCherry, respectively (Figures 2B,D and Supplementary Table 2). Hence, these likely reflect freely diffusing mCherry-Smn and eGFP-Smn monomers. The second diffusion coefficient D_2_ (Figures 2C,E) is about 50–100 times lower than the first component. Similar to what was suggested for the observations in cell culture, these slow components likely represent Smn as oligomers or bound in a complex, possibly the SMN complex. It should be noted that there is a high heterogeneity within each pool of fast and slow components, as visible by large standard deviations, as reported in the Supplementary Tables, which reflects both the variability in the size of the protein aggregates and the heterogeneity of the intracellular environment.

Notably, we did not observe any dynamics of Smn that would be suggestive of active transport, neither in axons nor in the soma. On very few occasions (2 out of 38 measurements), we observed spikes in the intensity trace for mCherry-Smn, which are indicative of large particles passing through the confocal volume (Supplementary Figure 2B). However, similar spikes were also observed for mCherry (Supplementary Figure 2A). These intensity traces translated into ACFs that could not be fitted to 3D diffusion models (Supplementary Figures 2C,D, respectively). No such spikes were detected for eGFP or eGFP-Smn, suggesting that these spikes are caused by mCherry aggregates. Furthermore, the rate of movement of the particle was variable, and thus did not correspond to active transport. As a positive control, we used FCS to measure the flow of blood cells in zebrafish to demonstrate that FCS is able to differentiate between active transport and diffusion dynamics (Supplementary Figure 3). Measurements were taken in the region of blood flow into the yolk of the zebrafish and the experimental ACF fitted to a diffusion and flow model. As shown in Supplementary Figure 3A, two other models, the flow model and 3D 1-particle diffusion model did not fit well to the experimental ACF and hence were rejected. The velocity of the blood flow obtained (54.5 ± 6.1 μm/s) agrees with previous published data (Shi et al., 2009). Hence, FCS measurements allow us to observe the dynamics of Smn proteins and showed that mCherry-Smn diffuses in axons of motor neurons as monomeric and oligomeric form instead of being actively transported.

### Dynamics of Smn in Motor Neurons Is Affected Under Knock-Down Conditions

As transgenic *hb9*:mCherry-Smn embryos also expressed Smn endogenously, Smn oligomers consisted of both endogenous and FP tagged Smn. Hence, we next studied Smn dynamics when endogenous Smn levels were knocked-down by Morpholino oligos (MOs). These MOs target the ATG region and efficiently block translation of endogenous *smn* mRNA as described previously (McWhorter et al., 2003; Winkler et al., 2005). Transgenic mCherry-Smn was not knocked down, as it contained five wobble bases in the *smn* ATG region. We hypothesized that by knocking down endogenous Smn, mCherry-Smn would replace their binding sites in the complexes, which would then affect the dynamics of the diffusional components.

To show that mCherry-Smn has not been knocked down and is still functional, a rescue experiment was carried out (Supplementary Figure 4). The Morphologies of seven caudal primary axons above the yolk extension region for both rows of motor neurons in the region were analyzed in each larva at 31 hpf. The mean percentage of normal, branched and truncated axons are plotted in Supplementary Figure 4F. *smn* morpholino knock-down in Wildtype (WT) zebrafish resulted in defective axons, which showed extensive branching and some truncation (Supplementary Figure 4B), compared to uninjected controls, or WT injected with control MO (Supplementary Figure 4A). The axonal defects were partially rescued when the *smn* morpholino were injected into transgenic *hb9*-mCherry-Smn embryos (Supplementary Figure 4C) and were almost fully recovered when *mCherry-smn* mRNA was injected into the embryo (Supplementary Figure 4D). Thus, mCherry-Smn is functional and not affected by the presence of the tagged fluorescent protein.

In the *smn MO* knockdown experiment, we observed that a deficiency in endogenous Smn affected the dynamics of Smn in cell bodies and axons differently. This is likely because Smn exists in different types of complexes in the cell body as compared to the axons.

**FIGURE 3:**
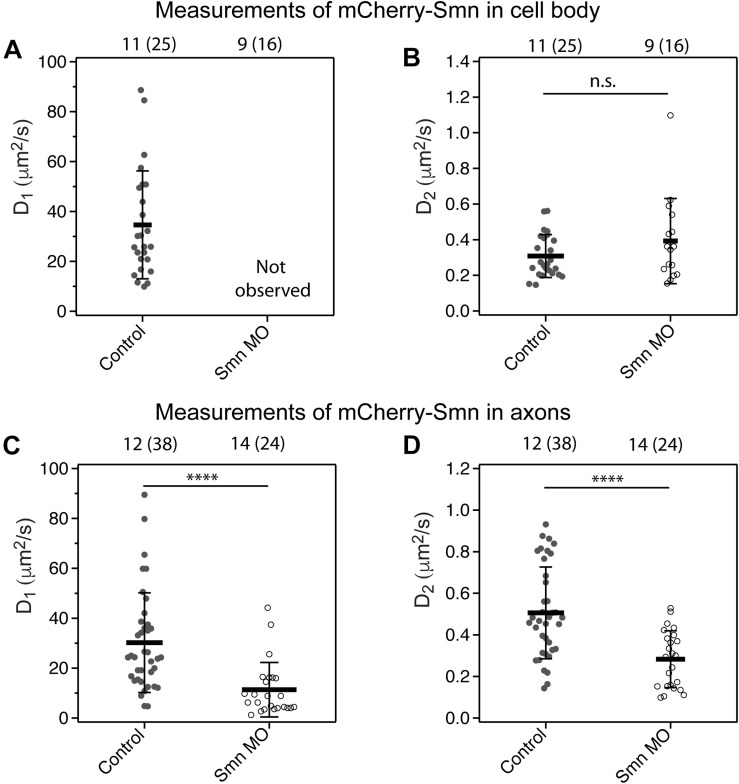
Diffusion coefficient of mCherry-Smn in motor neurons under *smn* MO conditions. **(A,B)** Average diffusion coefficients, D_1,_ D_2_ (μm^2^/s) of mCherry-Smn in motor neuron cell bodies under uninjected control and *smn* MO conditions. D_1_ was not observed for mCherry-Smn under *smn* MO conditions. **(C,D)** Average diffusion coefficients, D_1,_ D_2_ (μm^2^/s) of mCherry-Smn in axons for uninjected control and *smn* MO conditions. Error bars represent the SD. (^*n.s.*^*P* > 0.05, ^****^P < 0.0001). The numbers above the graph indicate the number of fish (number of points) the measurements were taken in.

In the cell body under Smn deficient conditions, ACFs obtained from measurements fitted to 3D 1-particle diffusion model with a diffusion coefficient similar to the slow component only (Figures 3A,B). We inferred that under Smn knockdown conditions, diffusible monomeric mCherry-Smn could have taken the place of endogenous Smn in the larger complexes or oligomers, as they are functional. This resulted in a depletion of monomeric mCherry-Smn such that they were no longer observable (Figure 3A) and only the slow component is being observed in the cell body (Figure 3B).

In axons, on the other hand, the dynamics of Smn under knock-down conditions was affected differently. The ACFs obtained could still be fitted to 3D 2-particle diffusion models (Figure 3C,D). However, there was an overall slow-down in diffusion of both the fast and slow components. The diffusion coefficient of the fast component, D_1_, was a third lower than that of uninjected controls, accompanied by an increase in its fraction, F_1_ (F_1_ = 1−F_2_) (Supplementary Table 3). The diffusion of the complexes (Figure 3D) may be slowed down due to the presence of the additional tagged fluorescent proteins if the complex involves a substantial number of Smn proteins. The components in Figure 3C could represent a separate set of smaller complexes previously not observable under a WT condition, however, more studies will be needed to investigate the compositions or nature of these complexes.

Overall, these findings suggest that the slow component in the diffusional models consists of oligomers of Smn.

### Dynamics of Truncated Smn Differs From Full Length Smn

We next tried to differentiate further between the two components of Smn. Exon 7 is important for localization of SMN to axons while exon 6 is important for the dimerization of SMN (Lorson et al., 1998). The plasmid *hb9*:eGFP-SmnΔex6,7 was generated for expression of an eGFP-Smn that is truncated at the end of exon 5. We expected that the dynamics of truncated eGFP-Smn would be faster than that of full length Smn as oligomerization is affected.

**FIGURE 4:**
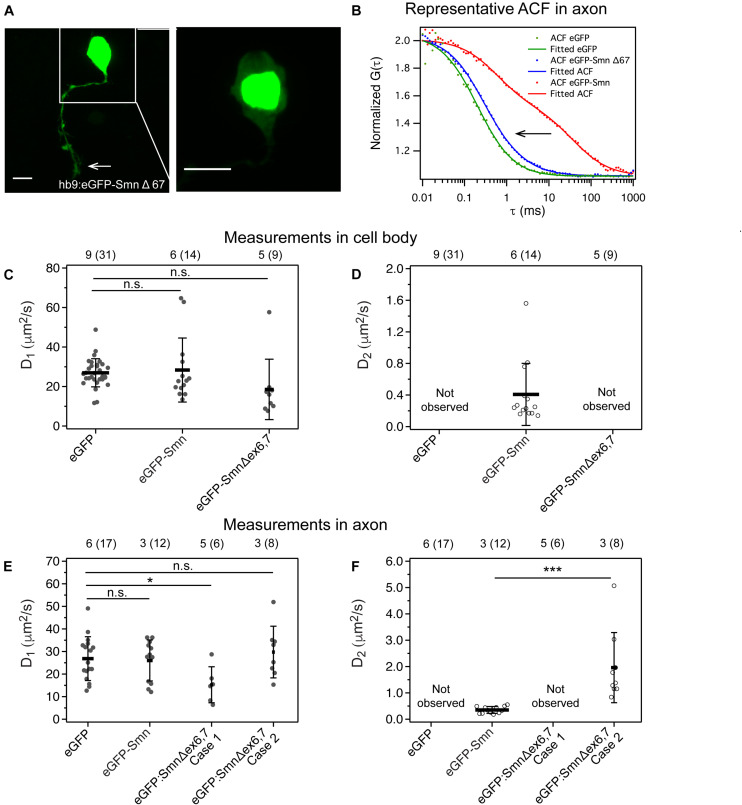
Expression and dynamics of eGFP- Smn Δex6,7 in zebrafish motor neurons. **(A)** Expression of eGFP- SmnΔex6,7 in motor neuron cell body and axon at 2 dpf. It is expressed strongly in the nucleus and weakly in axon and cytoplasm of cell body. **(B)** Representative ACF and fitted ACF of eGFP, eGFP-Smn and eGFP-SmnΔex6,7. **(C)** Average diffusion coefficients, D_1_ (μm^2^/s) of eGFP, eGFP-Smn, eGFP-SmnΔex6,7 in cell body. **(D)** Average diffusion coefficients, D_2_ (μm^2^/s) of eGFP-Smn, eGFP-SmnΔex6,7 in cell body. **(E)** Average diffusion coefficients, D_1_ (μm^2^/s) of eGFP, eGFP-Smn, eGFP- SmnΔex6,7 in axons. **(F)** Average diffusion coefficients, D_2_ (μm^2^/s) of eGFP-Smn, eGFP-SmnΔex6,7 in axons. Error bars represent the SD. (^*n.s.*^*P* > 0.05, ^∗^*P* < 0.05, ^∗∗∗^*P* < 0.001). Measurements grouped under Case 1 were fitted to 3D 1-particle diffusion model, found in 6 out of 14 measurements, indicating a loss of the second component. Case 2 refers to measurements fitted to 3D 2-particle diffusion model, found in 8 out of 14 measurements. The numbers above the graph indicate the numbers of fish (number of points) the measurements were taken in.

We found that eGFP-SmnΔex6,7, in contrast to full-length eGFP-Smn, was strongly expressed in the nucleus and weakly in the cytoplasm of the cell body (Figure 4A, close-up image of cell body). Although exon 7 was reported to affect localization of Smn to axons (Zhang et al., 2003), we were still able to observe strong eGFP fluorescence in the axons (white arrow).

More interestingly, FCS measurements showed that the slow component, which was consistently detected with full-length eGFP-Smn, was no longer present. In the cytoplasm of the cell body, ACFs fitted to a 3D 1-particle diffusion model with a diffusion coefficient similar to that of eGFP and the fast component of eGFP-Smn (Figures 4C,D). This indicated that eGFP-SmnΔex6,7 was not able to oligomerize or bind to complexes, and instead diffused freely in the soma. In axons, there were two outcomes for the fitting, a 3D 1-particle diffusion model fit (indicated as Case 1) and a 3D 2-particle diffusion model fit (Case 2). Supplementary Figure 5 shows the comparison between a 3D 1-particle and 2-particle model fitting of representative ACFs in Case 1 and Case 2 and their respective residuals. The sole component in the 1-particle model and the fast component in the 2-particle model are in the same order of magnitude as the fast component of eGFP-Smn and eGFP, suggesting the presence of freely diffusing eGFP-SmnΔex6,7 in axons (Figure 4E and Supplementary Table 4). The diffusion coefficient of case 1 is slightly lower than that of eGFP. It is possible that these measurements are actually similar to case 2 only that the slow component is so low in fraction that we cannot distinguish two components (Meseth et al., 1999). In cases of 2-particle fits, the slow component is much faster than the slow component of full-length eGFP-Smn (Figure 4E). There is a shift in the ACF to a shorter characteristic time for the second component of eGFP-SmnΔex6,7 (Figures 4B,F). The characteristic time in the ACF is inversely proportional to diffusion coefficient (see Equation 2), while diffusion coefficient is inversely proportional to molecular weight. Particles with lower molecular weight will diffuse faster and have a shorter characteristic time in ACF. Hence this suggests that the particle that is associated with truncated eGFP-SmnΔex6,7 is of lower molecular weight than the complex formed by full-length Smn.

## Discussion

It has been hypothesized that SMN has a motor neuron specific role in axonal transport and controls localization of mRNAs important for axon outgrowth, neuromuscular junction (NMJ) maturation and maintenance (Fallini et al., 2012). However, studies analyzing the subcellular distribution of SMN in motor neurons are scarce. By quantitative analysis of Smn behavior in zebrafish motor neurons using FCS, we intended to provide more insight into its axonal function.

Fluorescence correlation spectroscopy is a technique with single-molecule sensitivity and allows the *in vivo* study of protein dynamics in motor neurons of zebrafish embryos. There are some limitations of FCS such as the need for a fluorescent label for the detection of the mobile particle. To differentiate between monomers and dimers, they need to be different in size by at least a factor of four and to distinguish between two different species, their diffusion coefficient has to be at least 1.6 times different (Meseth et al., 1999). However, the strength of FCS lies in its ability to measure and differentiate between diffusion and transport and to identify different types of species with its distinct dynamics from the fitting models.

In this study, we found that fluorescent protein-tagged Smn (FP-Smn) exists as two distinct components in both the cytoplasm of the cell body and axons. These Smn components represent a fast diffusing particle and a slow diffusing particle. What gives rise to the presence of two types of particles with different dynamics, and are the particles transported in the axons?

**FIGURE 5:**
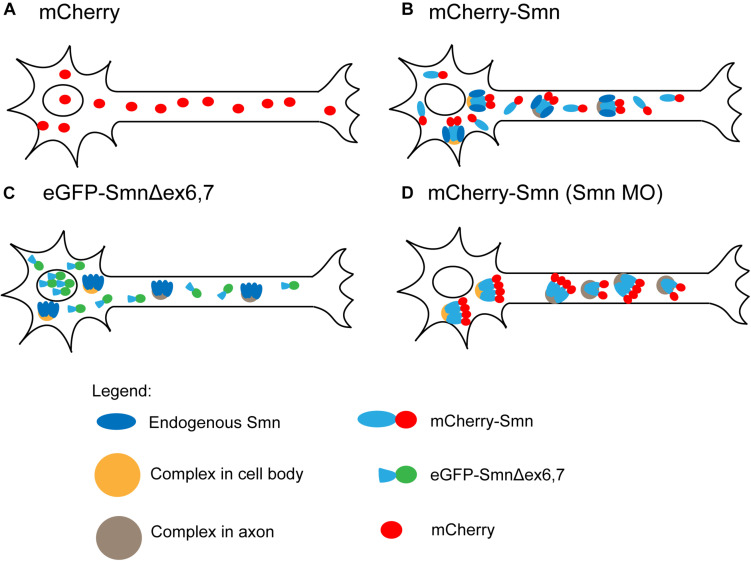
Summary of FCS observations. **(A)** Freely diffusing mCherry. **(B)** Two components of freely diffusing mCherry-Smn and mCherry-Smn with endogenous Smn in complexes that are distinct for cell body and axon (oligomers of mCherry-Smn and Smn are indicated by tetramers). **(C)** Truncated eGFP-Smn concentrates in nucleus, freely diffuses in cytoplasm. Complexes consist of only endogenous Smn. **(D)** Depletion of endogenous Smn led to a depletion of freely diffusing mCherry-Smn and mCherry-Smn replacing endogenous Smn in complexes. For details, see main text.

mCherry and eGFP were controls and diffused freely in motor neurons with a single diffusion coefficient, which reflects their monomeric nature (Figure 5A). The diffusion coefficient of the fast component of FP-Smn is in the same order of magnitude, hence this is likely a freely diffusing, monomeric form of FP-Smn. This was surprising as SMN protein rapidly oligomerizes (Pellizzoni et al., 2002) and associates with Gemin2–8 and UNR-interacting Protein (UNRIP) to form a large, stable, multi-subunit complex known as the SMN complex (Cauchi, 2010). Accordingly, we speculated that the slow component comprises Smn in an oligomeric and complex form. FCS data obtained in SH-SY5Y cells supported this. mCherry-Smn particles were brighter than mCherry and competitive binding existed between mCherry-Smn and untagged Smn, indicating the presence of mCherry-Smn oligomers. Hence, in a transgenic background, endogenous Smn and transgenic FP-Smn co-exist in oligomers in the complex. The presence of a monomeric FP-Smn form, represented by the fast component, could be due to the overexpression strategy used, thus excess FP-Smn is not associated in a complex.

The observation of large standard deviations for the diffusion coefficient of FP-Smn was expected considering the heterogeneity of the particles. The exact stoichiometry of members in the SMN complex is unknown (Gupta et al., 2015). Sucrose gradient centrifugation showed that SMN complexes are heterogeneously dispersed macromolecular aggregates ranging in size from 20S to 80S (Paushkin et al., 2002). It is difficult to pinpoint the exact species that makes up the slow component as SMN associates with a wide range of proteins or protein complexes involved in translation, transcription or RNA metabolism (reviewed by Rossoll and Bassell, 2009), or with components of the SMN complex. Based on our FCS data, we speculate that the slowly diffusing components represent SMN complexes involved in snRNP assembly, as well as Smn, likely in an oligomeric form that is associated with various proteins (Figure 5B), which is in agreement with recent findings (Veepaschit et al., 2021). We speculate that two pools of FP-Smn exist in motor neurons, one that is monomeric and another that oligomerizes with endogenous Smn, bound to complexes.

Both the expression and dynamics of Smn were affected when exons 6 and 7 were deleted. The accumulation of eGFP-SmnΔex6,7 in nuclei is consistent with a previous report that also showed enrichment of eGFP-SMNΔex7 in the nuclei of chick forebrain neurons (Zhang et al., 2003). It was suggested that a sequence of five amino acids, QNQKE, in exon 7 contains a cytoplasmic localization signal that when deleted results in nuclear accumulation of Smn protein (Zhang et al., 2003). However, while that report showed that axons were completely devoid of eGFP-SMNΔex7, weak expression of eGFP-SmnΔex6,7 could be observed in the axons here. This raises the important question whether the dynamics of this axonal pool of eGFP-SmnΔex6,7 differs from that of full length Smn.

Interestingly, FCS measurements of eGFP-SmnΔex6,7 in cell bodies no longer detected the slow component that represented complexes formed by full length Smn. Instead, a single diffusing component, with diffusion coefficient in the same order of magnitude as eGFP, was observed. In axons, on the other hand, ∼50% of the measurements revealed a single fast diffusing component and the other ∼50% showed a fast component and a slow component. However, this slow component was much faster than that observed for full-length Smn. Exons 2b and 6 were previously reported to be required for dimerization and oligomerization of SMN (Lorson et al., 1998; Young et al., 2000). SMN lacking exon 7 also loses its ability to self-oligomerize (Lorson et al., 1998; Pellizzoni et al., 1999). Hence Smn, lacking exons 6 and 7, is defective in oligomerization and cannot be incorporated into the SMN complex. Together, this reinforces the speculation that the slow component is Smn in oligomeric form, bound to complexes. eGFP-SmnΔex6,7 is concentrated in the nucleus (Figure 5C). Particles present in cell body and axons are single diffusing particles, while complexes only consist of endogenous Smn without eGFP-SmnΔex6,7.

Next, knocking down endogenous Smn levels had different effects on the behavior of mCherry-Smn in cell body and axons. In the cell body, only a single slow diffusing particle was observed, while two types of particles were observed in axons, both of which were much slower than those under WT condition (Figure 5D). We speculate that as the endogenous pool of Smn is knocked-down, freely diffusing monomeric mCherry-Smn replaces their binding sites in the complexes, hence depleting the pool of freely diffusing monomeric mCherry-Smn such that they were no longer observable and only the slow component is being observed in the cell body.

As the effect on diffusion is different in cell body and the axons, our data suggest that mCherry-Smn exists in distinct types of complexes that are different in cell body and axons. As mCherry-Smn takes the place of endogenous Smn in some of the complexes, the diffusion of the complexes may be slowed down in the axons due to the presence of the additional FP-tag if the complex involves a substantial number of Smn proteins. Earlier reports showed that SMN granules in neurites of primary motor neurons (Zhang et al., 2006) and in differentiated human neuroblastoma cells (Todd et al., 2010) do not contain essential snRNP components such as Sm proteins. This suggests that the axonal pool of SMN that we detected by FCS could be involved in a role different from snRNP assembly and have different requirements for exons 6 and 7.

It is an ongoing controversial debate whether SMN has a motor neuron specific function, other than snRNP assembly, that is responsible for selective motor neuron vulnerability in SMA patients. Detection of axonal transport would have helped to settle this debate. However, an ACF that could be fitted to an active flow model, which is expected if active transport of Smn occurs, was not obtained. On rare occasions, spikes in the intensity trace, indicative of large particles passing through the confocal volume, were observed (Supplementary Figure 2). However, such occurrences were infrequent and the characteristic time of the dynamics was variable so that this could not be attributed to a real transport process. We conclude that the type of FP (mCherry or eGFP) overall does not influence the behavior of Smn in motor neurons, albeit for presence of rare mCherry aggregates. In addition, transiently expressed proteins and stably expressed proteins do not differ in their dynamics or expression. Smn, in both cell bodies and axons, exists as a freely, fast diffusing and a slow diffusing, complex-bound particle, and there is no indication of active transport in the axons.

We have consistently observed diffusional dynamics of mCherry-Smn at various locations and time points (48 hpf, see text; and 31 hpf, data not shown) along the axons and hence also hypothesized that these Smn particles have traveled to the axons from the cell body through a diffusion process. As a systematic study of the dynamics of mCherry-Smn at the specific entry point of the axons from the cell body has not be carried out, we cannot exclude that the first mCherry-Smn to appear in the axons could have been actively transported from the soma to the entry point into the axon. However, it should be noted that given a diffusion coefficient of 1 or 30 μm^2^/s, the root mean squared displacement of an Smn protein is ∼1.5 or ∼7.7 μm in a second. This would in principle allow complete diffusion throughout the axons in a short time.

In a previous report, antero- and retrograde movement of GFP tagged SMN was described for chick forebrain primary neurons in the form of granular particles (Zhang et al., 2003). SMN-granules, on its own or co-localized with Gemin2, or mRNA binding proteins, HuD and IMP1, were reported to move along axons in primary motor neurons, with a proportion of granules oscillating within a localized region (Fallini et al., 2010, 2011, 2014). We observed movement of granule-like particles by time-lapse confocal imaging not only of mCherry-Smn, but also mCherry itself, transiently expressed in motor axons of 2 dpf zebrafish (data not shown). Hence, such granule-like particles are unlikely to be specific to mCherry-Smn and may be attributed to an artifact created by the aggregation and movement of the fluorescent protein itself. FCS was able to measure GFP, presumably accumulated in vesicles, actively transported along tubular structures in plants in the form of ‘pulsed’ transport of bright batches (Kohler et al., 2000). So far, none of our FCS measurements, which have a much higher sensitivity than confocal imaging, provides any support for the hypothesis that Smn is transported in motor axons.

Remarkably, a recent report suggested that SMN acts as a molecular chaperone for the assembly of messenger ribonucleoprotein (mRNP) complexes in axons, analogous to its role in snRNP assembly (Donlin-Asp et al., 2017). Reduction in the association of IMP1 with β-*actin* mRNA in a SMA mouse model and fibroblasts derived from SMA patients was observed. Therefore, the authors suggested that SMN is important for the assembly of IMP1 protein with β-*actin* mRNA via a transient association. In our zebrafish assay, the slow diffusing component observed in our FCS studies, both in axons and the cell body could represent a wide range of protein complexes associated with Smn, and Smn transiently associated with mRNP complexes could possibly be one of the diffusional particles. Hence, our observations made by FCS in motor neurons *in vivo* are in agreement with the findings by Donlin-Asp et al. (2017).

## Conclusion

Our *in vivo* FCS approach identified the existence of oligomers and suggested the presence of different complexes in axons and cell bodies but did not observe any axonal transport.

## Data Availability Statement

The original contributions generated for this study are included in the article/Supplementary Material, further inquiries can be directed to the corresponding authors.

## Ethics Statement

The animal study was reviewed and approved by National University of Singapore (NUS) Institutional Animal Care and Use Committee (IACUC).

## Author Contributions

AK performed the FCS measurements, MVS performed the rescue experiments, JO performed the *in vitro* oligomerization studies. UF, CW, and TW conceived the project. AK, CW, and TW wrote the manuscript. All authors edited and agreed to the manuscript content.

## Conflict of Interest

The authors declare that the research was conducted in the absence of any commercial or financial relationships that could be construed as a potential conflict of interest.

## Publisher’s Note

All claims expressed in this article are solely those of the authors and do not necessarily represent those of their affiliated organizations, or those of the publisher, the editors and the reviewers. Any product that may be evaluated in this article, or claim that may be made by its manufacturer, is not guaranteed or endorsed by the publisher.

## Supplementary Material

Supplementary Figure 1 *In vitro* oligomerization of zebrafish and human SMN. **(A–C)** Gel filtration traces of purified GST-*h*SMN(252-284) **(A)**, purified His_6_-MBP-*zf*SMN*wt* **(B)**, and a mixture of both **(C)**. **(A′–C′)** Analyses by SDS-PAGE, visualized by Coomassie staining of the indicated fractions from the respective traces. The human-zebrafish hybrid complex **(C,C′)** displays very similar oligomerization properties as the human- **(A,A′)** and zebrafish-only controls **(B,B′)**.

Supplementary Figure 2 Intensity traces of mCherry and mCherry-Smn that showed spikes and their respective ACF. **(A)** Intensity trace of 10 s out of a 30 s FCS measurement of mCherry, showing a spike in the intensity. **(B)** 20 s out of a 120 s FCS measurement of mCherry-Smn, showing spikes in the intensity. **(C)** ACF of the mCherry intensity trace, which shows a step at the end that cannot be fitted to a 3D diffusion model. **(D)** ACF of the mCherry-Smn intensity trace that cannot be fitted to a 3D diffusion model.

Supplementary Figure 3 FCS measurement of blood flow in zebrafish. **(A)** Representative ACF for blood flow in zebrafish fitted to three different fitting models, Diffusion and Flow Model, Flow Model and 3D 1-particle diffusion model. The experimental ACF fitted to a Diffusion and Flow model, the other two fitting models were rejected. **(B)** Velocity of the blood flow. Mean ± SD (SEM) = 54.5 ± 18.2 (6.1) μm/s. Error bars represent the SD. The numbers above the graph indicate the numbers of fish (number of points) the measurements were taken in.

Supplementary Figure 4 mCherry-Smn can rescue axonal defects in *smn* morpholino knocked-down embryos. **(A–D)** Representative images of Znp-1 immunostained embryos at 31 hpf. Scale bar = 100 μm. **(A)** WT embryos injected with control MO. The axons are normal without any branching or truncation. **(B)** WT embryos injected with *smn* MO. Significant number of axons are branched or truncated. **(C)** *hb9*:mCherry-Smn transgenic line injected with *smn* MO. Axonal defects partially rescued by mCherry-Smn. **(C′)** Imaging of mCherry fluorescence present only in motor neurons. **(D)** WT embryos injected with *smn* MO and 100 ng/μL of *mCherry-smn* mRNA. **(D′)** Imaging of mCherry fluorescence ubiquitously present. **(E)** Western blot analysis, showing that endogenous Smn protein is present in uninjected control and reduced in *smn* MO knock-down samples. mCherry-Smn protein is present in sample injected with *mCherry-smn* mRNA. **(F)** Mean percentage of normal, branched and truncated axons across three biological replicates (comprising n number of fish analyzed in total with 14 axons analyzed in each fish). Error bars represent the SD of the three replicates. Statistical analyses were done using one-way ANOVA test.

Supplementary Figure 5 Comparison of the fitting models in Case 1 and Case 2. **(A,B)** Representative ACFs for Case 1 and Case 2 fitted to both 3D 1-particle and 2-particle diffusion models. **(C,D)** Residuals for a 3D 1-particle diffusion model fit. **(E,F)** Residuals for a 3D 2-particle diffusion model fit. In Case 1, the fit quality shown by the residuals indicates that a 3D 1-particle fit was sufficient and a 2-particle fit did not improve the fitting quality considerably. Moreover, non-physical parameters were obtained for the 2-particle fit (negative fractions, non-consistent widely varying diffusion coefficients). In Case 2, a 2-particle diffusion model improved the fitting quality and thus was chosen.

Supplementary Table 1 Mean diffusion coefficients, D_1_, D_2_ and fraction of second components of mCherry, mCherry-Smn, mCherry-Smn: untagged Smn in cytoplasm and nucleus of SH-SY5Y cells.

Supplementary Table 2 Diffusion coefficients, D_1_, D_2_ and fraction of second components, F_2_ for FCS measurements in cell body and axon.

Supplementary Table 3 Diffusion coefficients, D_1_, D_2_ and fraction of second components, F_2_ for FCS measurements of mCherry-Smn under *smn* MO conditions. Data for uninjected control is taken from mCherry-Smn in Supplementary Table 2.

Supplementary Table 4 Diffusion coefficients of eGFP-SmnΔex6,7 compared to eGFP and eGFP-Smn in the cell body and axon. Data of eGFP and eGFP-Smn are taken from Supplementary Table 2. Measurements grouped under Case 1 were fitted to 3D 1-particle diffusion model, found in 6 out of 14 measurements, indicating a loss of the second component. Case 2 refers to measurements fitted to 3D 2-particle diffusion model, found in 8 out of 14 measurements.

## References

[B1] Bechade C. Rostaing P. Cisterni C. Kalisch R. La Bella V. Pettmann B. (1999). Subcellular distribution of survival motor neuron (SMN) protein: possible involvement in nucleocytoplasmic and dendritic transport. *Eur. J. Neurosci.* 11 293–304. 10.1046/j.1460-9568.1999.00428.x 9987032

[B2] Burghes A. H. M. Beattie C. E. (2009). Spinal muscular atrophy: why do low levels of survival motor neuron protein make motor neurons sick? *Nat. Rev. Neurosci.* 10 597–609. 10.1038/nrn2670 19584893 PMC2853768

[B3] Cauchi R. J. (2010). SMN and gemins: ‘we are family’. or are we?: insights into the partnership between gemins and the spinal muscular atrophy disease protein SMN. *Bioessays* 32 1077–1089. 10.1002/bies.201000088 20954180

[B4] Chari A. Golas M. M. Klingenhager M. Neuenkirchen N. Sander B. Englbrecht C. (2008). An assembly chaperone collaborates with the SMN complex to generate spliceosomal SnRNPs. *Cell* 135 497–509. 10.1016/j.cell.2008.09.020 18984161

[B5] Chari A. Paknia E. Fischer U. (2009). The role of RNP biogenesis in spinal muscular atrophy. *Curr. Opin. Cell Biol.* 21 387–393. 10.1016/j.ceb.2009.02.004 19286363

[B6] Donlin-Asp P. G. Fallini C. Campos J. Chou C. C. Merritt M. E. Phan H. C. (2017). The survival of motor neuron protein acts as a molecular chaperone for mRNP assembly. *Cell Rep.* 18 1660–1673. 10.1016/j.celrep.2017.01.059 28199839 PMC5492976

[B7] Fallini C. Bassell G. J. Rossoll W. (2010). High-efficiency transfection of cultured primary motor neurons to study protein localization, trafficking, and function. *Mol. Neurodegener* 5:17. 10.1186/1750-1326-5-17 20406490 PMC2867961

[B8] Fallini C. Bassell G. J. Rossoll W. (2012). Spinal muscular atrophy: the role of SMN in axonal mRNA regulation. *Brain Res.* 1462 81–92. 10.1016/j.brainres.2012.01.044 22330725 PMC3360984

[B9] Fallini C. Rouanet J. P. Donlin-Asp P. G. Guo P. Zhang H. Singer R. H. (2014). Dynamics of survival of motor neuron (SMN) protein interaction with the mRNA-binding protein IMP1 facilitates its trafficking into motor neuron axons. *Dev. Neurobiol.* 74 319–332. 10.1002/dneu.22111 23897586 PMC3906212

[B10] Fallini C. Zhang H. Su Y. Silani V. Singer R. H. Rossoll W. (2011). The survival of motor neuron (SMN) protein interacts with the mRNA-binding protein HuD and regulates localization of poly(A) mRNA in primary motor neuron axons. *J. Neurosci.* 31 3914–3925. 10.1523/jneurosci.3631-10.2011 21389246 PMC3070748

[B11] Fan L. Simard L. R. (2002). Survival motor neuron (SMN) protein: role in neurite outgrowth and neuromuscular maturation during neuronal differentiation and development. *Hum. Mol. Genet.* 11 1605–1614. 10.1093/hmg/11.14.1605 12075005

[B12] Feldkotter M. Schwarzer V. Wirth R. Wienker T. F. Wirth B. (2002). Quantitative analyses of SMN1 and SMN2 based on real-time lightCycler PCR: fast and highly reliable carrier testing and prediction of severity of spinal muscular atrophy. *Am. J. Hum. Genet.* 70 358–368. 10.1086/338627 11791208 PMC419987

[B13] Giavazzi A. Setola V. Simonati A. Battaglia G. (2006). Neuronal-specific roles of the survival motor neuron protein: evidence from survival motor neuron expression patterns in the developing human central nervous system. *J. Neuropathol. Exp. Neurol.* 65 267–277. 10.1097/01.jnen.0000205144.54457.a3 16651888

[B14] Glinka M. Herrmann T. Funk N. Havlicek S. Rossoll W. Winkler C. (2010). The heterogeneous nuclear ribonucleoprotein-R is necessary for axonal beta-actin mRNA translocation in spinal motor neurons. *Hum. Mol. Genet.* 19 1951–1966. 10.1093/hmg/ddq073 20167579

[B15] Gupta K. Martin R. Sharp R. Sarachan K. L. Ninan N. S. Van Duyne G. D. (2015). Oligomeric properties of survival motor neurongemin2 complexes. *J. Biol. Chem.* 290 20185–20199. 10.1074/jbc.m115.667279 26092730 PMC4536428

[B16] Hao L. T. Duy P. Q. Jontes J. D. Beattie C. E. (2015). Motoneuron development influences dorsal root ganglia survival and Schwann cell development in a vertebrate model of spinal muscular atrophy. *Hum. Mol. Genet.* 24 346–360. 10.1093/hmg/ddu447 25180019 PMC4275068

[B17] Kapusta P. (2010). *Absolute Diffusion Coefficients: Compilation of Reference Data for FCS Calibration.* Berlin: PicoQuant.

[B18] Kohler R. H. Schwille P. Webb W. W. Hanson M. R. (2000). Active protein transport through plastid tubules: velocity quantified by fluorescence correlation spectroscopy. *J. Cell Sci.* 113:3921. 10.1242/jcs.113.22.3921 11058079

[B19] Kye M. J. Niederst E. D. Wertz M. H. Gonçalves Ido C. Akten B. Dover K. Z. (2014). SMN regulates axonal local translation via miR-183/mTOR pathway. *Hum. Mol. Genet.* 23 6318–6331. 10.1093/hmg/ddu350 25055867 PMC4271102

[B20] Lauria F. Bernabò P. Tebaldi T. Groen E. J. N. Perenthaler E. Maniscalco F. (2020). SMN-primed ribosomes modulate the translation of transcripts related to spinal muscular atrophy. *Nat Cell Biol.* 22 1239–1251. 10.1038/s41556-020-00577-7 32958857 PMC7610479

[B21] Lefebvre S. Burglen L. Reboullet S. Clermont O. Burlet P. Viollet L. (1995). Identification and characterization of a spinal muscular atrophy-determining gene. *Cell* 80 155–165. 7813012 10.1016/0092-8674(95)90460-3

[B22] Liu Q. Dreyfuss G. (1996). A novel nuclear structure containing the survival of motor neurons protein. *EMBO J.* 15 3555–3565. 10.1002/j.1460-2075.1996.tb00725.x 8670859 PMC451956

[B23] Lorson C. L. Strasswimmer J. Yao J. M. Baleja J. D. Hahnen E. Wirth B. (1998). SMN oligomerization defect correlates with spinal muscular atrophy severity. *Nat. Genet.* 19 63–66. 10.1038/ng0598-63 9590291

[B24] McWhorter M. L. Monani U. R. Burghes A. H. Beattie C. E. (2003). Knockdown of the survival motor neuron (Smn) protein in zebrafish causes defects in motor axon outgrowth and pathfinding. *J. Cell Biol.* 162 919–931. 10.1083/jcb.200303168 12952942 PMC1761110

[B25] Meseth U. Wohland T. Rigler R. Vogel H. (1999). Resolution of fluorescence correlation measurements. *Biophys. J.* 76 1619–1631. 10.1016/s0006-3495(99)77321-2 10049342 PMC1300138

[B26] Mistri T. K. Devasia A. G. Chu L. T. Ng W. P. Halbritter F. Colby D. (2015). Selective influence of Sox2 on POU transcription factor binding in embryonic and neural stem cells. *EMBO Rep.* 16 1177–1191. 10.15252/embr.201540467 26265007 PMC4576985

[B27] Narayanan U. Ospina J. K. Frey M. R. Hebert M. D. Matera A. G. (2002). SMN, the spinal muscular atrophy protein, forms a pre-import snRNP complex with snurportin1 and importin beta. *Hum. Mol. Genet.* 11 1785–1795. 10.1093/hmg/11.15.1785 12095920 PMC1630493

[B28] Neuenkirchen N. Englbrecht C. Ohmer J. Ziegenhals T. Chari A. Fischer U. (2015). Reconstitution of the human U snRNP assembly machinery reveals stepwise Sm protein organization. *EMBO J.* 34 1925–1941. 10.15252/embj.201490350 26069323 PMC4547896

[B29] Pagliardini S. Giavazzi A. Setola V. Lizier C. Di Luca M. DeBiasi S. (2000). Subcellular localization and axonal transport of the survival motor neuron (SMN) protein in the developing rat spinal cord. *Hum. Mol. Genet.* 9 47–56. 10.1093/hmg/9.1.47 10587577

[B30] Paushkin S. Gubitz A. K. Massenet S. Dreyfuss G. (2002). The SMN complex, an assemblyosome of ribonucleoproteins. *Curr. Opin. Cell Biol.* 14 305–312. 10.1016/s0955-0674(02)00332-0 12067652

[B31] Pellizzoni L. Charroux B. Dreyfuss G. (1999). SMN mutants of spinal muscular atrophy patients are defective in binding to snRNP proteins. *Proc. Natl. Acad. Sci. U.S.A.* 96 11167–11172. 10.1073/pnas.96.20.11167 10500148 PMC18005

[B32] Pellizzoni L. Yong J. Dreyfuss G. (2002). Essential role for the SMN complex in the specificity of snRNP assembly. *Science* 298 1775–1779. 10.1126/science.1074962 12459587

[B33] Rembold M. Lahiri K. Foulkes N. S. Wittbrodt J. (2006). Transgenesis in fish: efficient selection of transgenic fish by co-injection with a fluorescent reporter construct. *Nat. Protoc.* 1 1133–1139. 10.1038/nprot.2006.165 17406394

[B34] Rossoll W. Bassell G. J. (2009). “Spinal muscular atrophy and a model for survival of motor neuron protein function in axonal ribonucleoprotein complexes,” in *Cell Biology of the Axon*, ed. Koenig E. (Berlin: Springer), 87–107. 10.1007/400_2009_4 PMC3718852 19343312

[B35] Rossoll W. Kroning A. K. Ohndorf U. M. Steegborn C. Jablonka S. Sendtner M. (2002). Specific interaction of Smn, the spinal muscular atrophy determining gene product, with hnRNP-R and gry-rbp/hnRNP-Q: a role for Smn in RNA processing in motor axons? *Hum. Mol. Genet.* 11 93–105. 10.1093/hmg/11.1.93 11773003

[B36] See K. Yadav P. Giegerich M. Cheong P. S. Graf M. Vyas H. (2014). SMN deficiency alters Nrxn2 expression and splicing in zebrafish and mouse models of spinal muscular atrophy. *Hum. Mol. Genet.* 23 1754–1770. 10.1093/hmg/ddt567 24218366

[B37] Shi X. Teo L. S. Pan X. Chong S. W. Kraut R. Korzh V. (2009). Probing events with single molecule sensitivity in zebrafish and Drosophila embryos by fluorescence correlation spectroscopy. *Dev. Dyn.* 238 3156–3167. 10.1002/dvdy.22140 19882725

[B38] Spiró Z. Koh A. Tay S. See K. Winkler C. (2016). Transcriptional enhancement of Smn levels in motoneurons is crucial for proper axon morphology in zebrafish. *Sci. Rep.* 6:27470. 27273160 10.1038/srep27470 PMC4895340

[B39] Todd A. G. Morse R. Shaw D. J. Stebbings H. Young P. J. (2010). Analysis of SMN-neurite granules: core cajal body components are absent from SMN-cytoplasmic complexes. *Biochem. Biophys. Res. Commun.* 397 479–485. 10.1016/j.bbrc.2010.05.139 20515655

[B40] Veepaschit J. Viswanathan A. Bordonné R. Grimm C. Fischer U. (2021). Identification and structural analysis of the Schizosaccharomyces pombe SMN complex. *Nucleic Acids Res.* 49 7207–7223. 10.1093/nar/gkab158 33754639 PMC8287938

[B41] Wang R. Brattain M. G. (2007). The maximal size of protein to diffuse through the nuclear pore is larger than 60kDa. *FEBS Lett.* 581 3164–3170. 10.1016/j.febslet.2007.05.082 17588566 PMC4064367

[B42] Winkler C. Eggert C. Gradl D. Meister G. Giegerich M. Wedlich D. (2005). Reduced U snRNP assembly causes motor axon degeneration in an animal model for spinal muscular atrophy. *Genes Dev.* 19 2320–2330. 10.1101/gad.342005 16204184 PMC1240041

[B43] Yamazaki T. Chen S. Yu Y. Yan B. Haertein T. C. Carrasco M. A. (2012). FUS-SMN protein interactions link the motor neuron diseases ALS and SMA. *Cell Rep.* 2 799–806. 10.1016/j.celrep.2012.08.025 23022481 PMC3483417

[B44] Young P. J. Man N. T. Lorson C. L. Le T. T. Androphy E. J. Burghes A. H. (2000). The exon 2b region of the spinal muscular atrophy protein, SMN, is involved in self-association and SIP1 binding. *Hum. Mol. Genet.* 9 2869–2877. 10.1093/hmg/9.19.2869 11092763

[B45] Zhang H. Xing L. Rossoll W. Wichterle H. Singer R. H. Bassell G. J. (2006). Multiprotein complexes of the survival of motor neuron protein SMN with Gemins traffic to neuronal processes and growth cones of motor neurons. *J. Neurosci.* 26 8622–8632. 10.1523/jneurosci.3967-05.2006 16914688 PMC4956918

[B46] Zhang H. L. Pan F. Hong D. Shenoy S. M. Singer R. H. Bassell G. J. (2003). Active transport of the survival motor neuron protein and the role of exon-7 in cytoplasmic localization. *J. Neurosci.* 23 6627–6637. 10.1523/jneurosci.23-16-06627.2003 12878704 PMC6740639

