# Supplementary material for: Fluorescence Correlation Spectroscopy Reveals Survival Motor Neuron Oligomerization but No Active Transport in Motor Axons of a Zebrafish Model for Spinal Muscular Atrophy
Source: Front Cell Dev Biol. 2021 Aug 11;9:639904. doi: 10.3389/fcell.2021.639904 (PMC8385639; doi:10.3389/fcell.2021.639904)
Supplement: Supplementary Figure 1 — In vitro oligomerization of zebrafish and human SMN. (A–C) Gel filtration traces of purified GST-hSMN(252-284) (A), purified His6-MBP-zfSMNwt (B), and a mixture of both (C). (A′–C′) Analyses by SDS-PAGE, visualized by Coomassie staining of the indicated fractions from the respective traces. The human-zebrafish hybrid complex (C,C′) displays very similar oligomerization properties as the human- (A,A′) and zebrafish-only controls (B,B′). [file Data_Sheet_1.pdf]

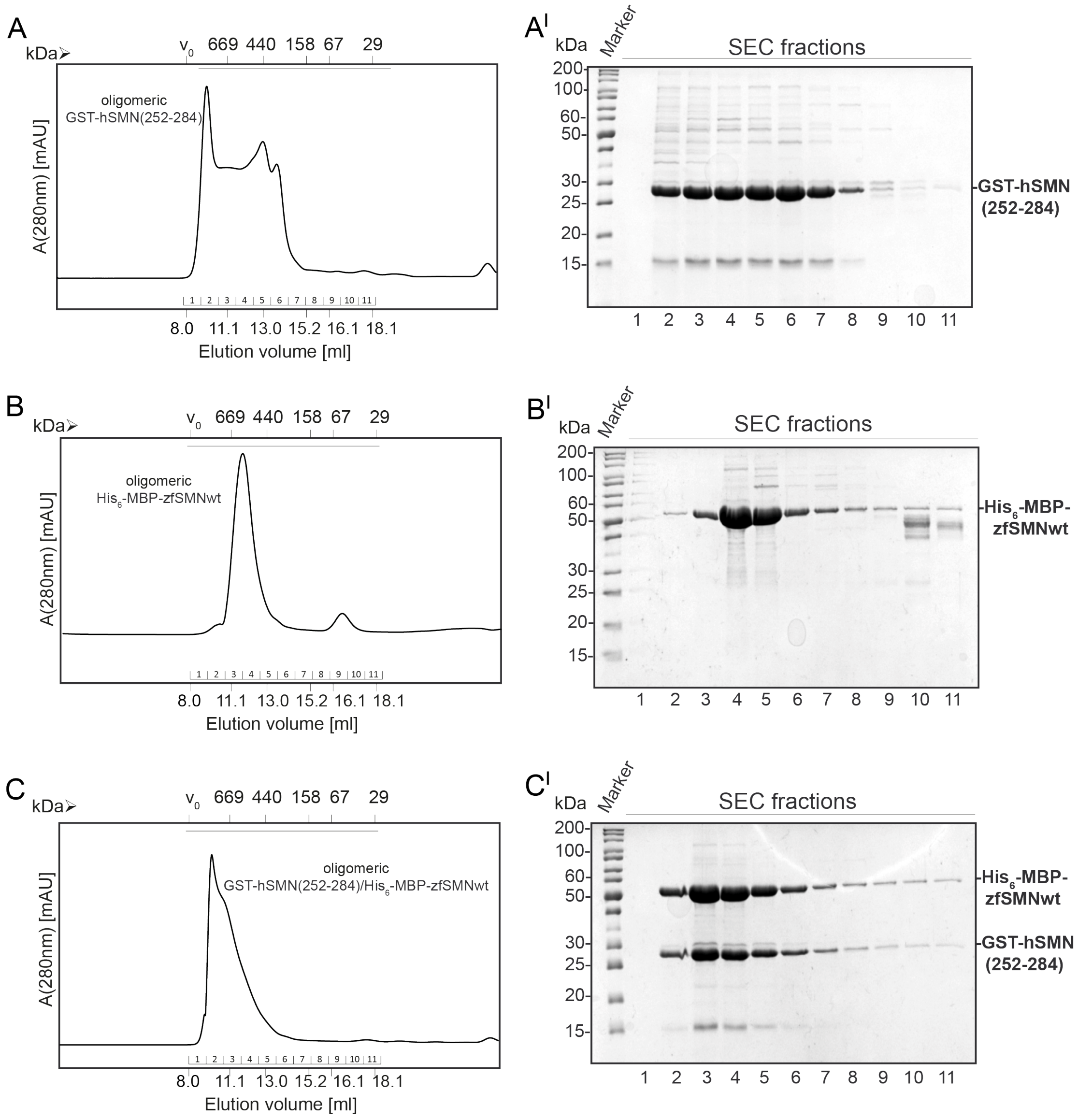

Figure S1. *In vitro* oligomerization of zebrafish and human SMN.

A, B, C. Gel filtration traces of purified GST-*h*SMN(252-284) (A), purified His_6-MBP-*zf*SMN*wt* (B), and a mixture of both (C). A', B', C'. Analyses by SDS-PAGE, visualized by Coomassie staining of the indicated fractions from the respective traces. The human-zebrafish hybrid complex (C, C') displays very similar oligomerization properties as the human- (A, A') and zebrafish-only controls (B, B').

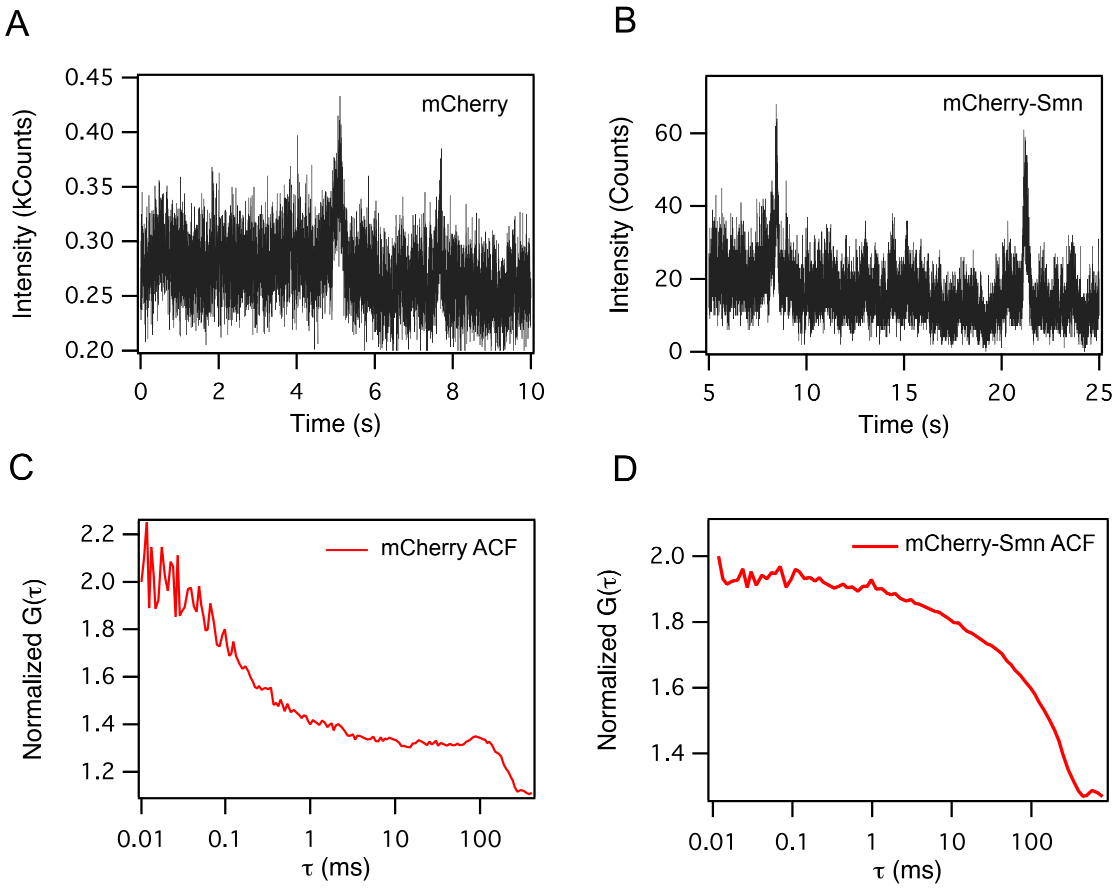

Figure S2. Intensity traces of mCherry and mCherry-Smn that showed spikes and their respective ACF.

A. Intensity trace of 10s out of a 30s FCS measurement of mCherry, showing a spike in the intensity. B. 20s out of a 120s FCS measurement of mCherry-Smn, showing spikes in the intensity. C. ACF of the mCherry intensity trace, which shows a step at the end that cannot be fitted to a 3D diffusion model. D. ACF of the mCherry-Smn intensity trace that cannot be fitted to a 3D diffusion model.

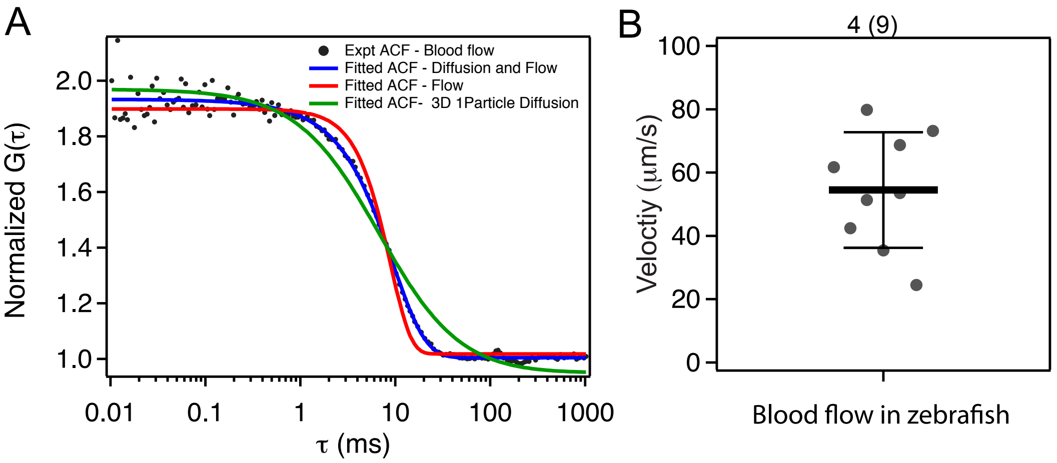

Figure S3. FCS measurement of blood flow in zebrafish

A. Representative ACF for blood flow in zebrafish fitted to three different fitting models, Diffusion and Flow Model, Flow Model and 3D 1-particle diffusion model. The experimental ACF fitted to a Diffusion and Flow model, the other two fitting models were rejected. B. Velocity of the blood flow. Mean ± SD (SEM) = 54.5 ± 18.2 (6.1) µm/s. Error bars represent the SD. The numbers above the graph indicate the numbers of fish (number of points) the measurements were taken in.

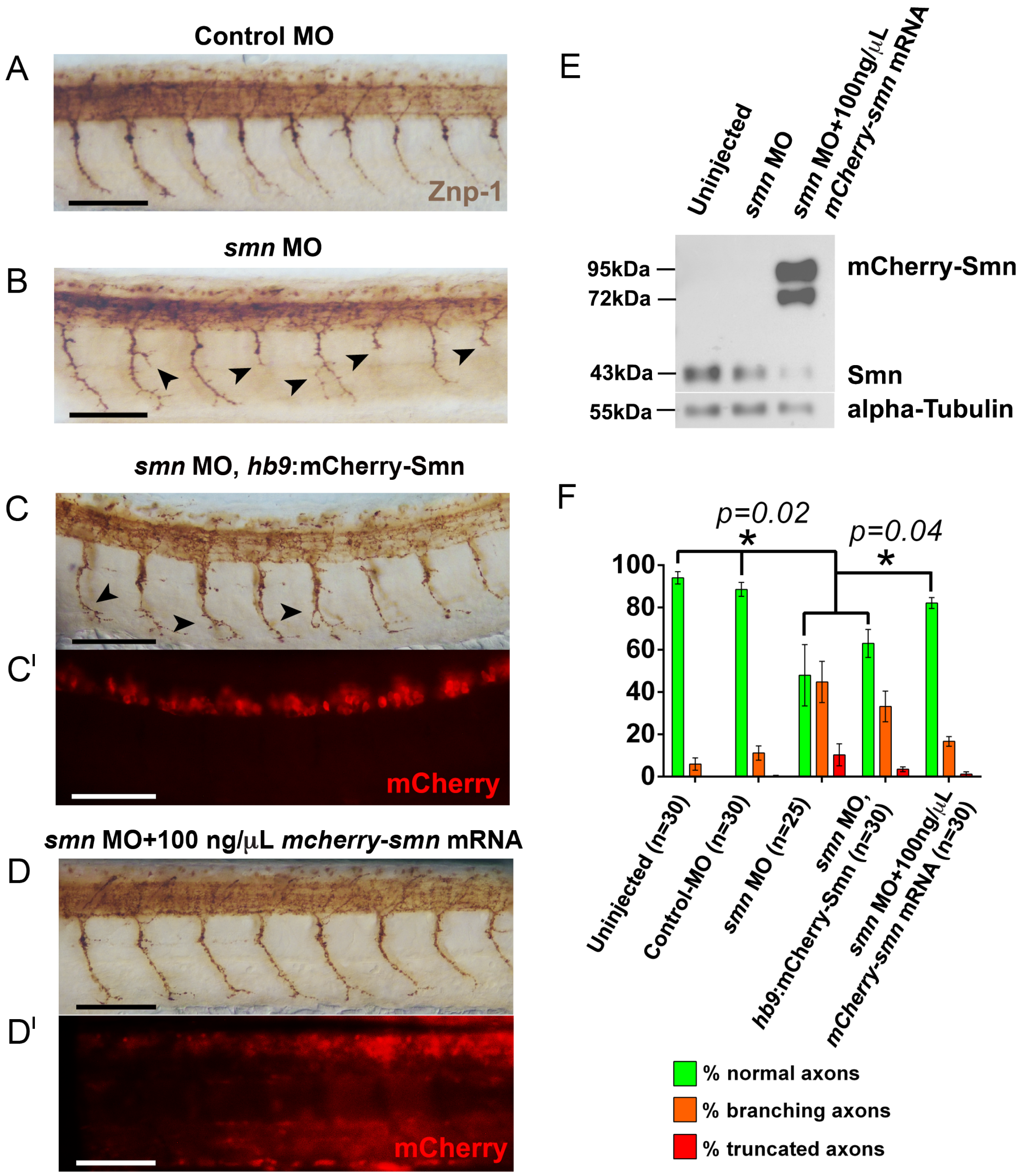

Figure S4 mCherry-Smn can rescue axonal defects in *smn* morpholino knocked-down embryos.

A-D. Representative images of Znp-1 immunostained embryos at 31hpf. Scale bar = 100 µm
A. WT embryos injected with control MO. The axons are normal without any branching or truncation. B. WT embryos injected with *smn* MO. Significant number of axons are branched or truncated. C. *hb9*:mCherry-Smn transgenic line injected with *smn* MO. Axonal defects partially rescued by mCherry-Smn. C'. Imaging of mCherry fluorescence present only in motor neurons. D. WT embryos injected with *smn* MO and 100ng/µL of *mCherry-smn* mRNA. D'. Imaging of mCherry fluorescence ubiquitously present. E. Western blot analysis, showing that endogenous Smn protein is present in uninjected control and reduced in *smn* MO knock-down samples. mCherry-Smn protein is present in sample injected with *mCherry-smn* mRNA. F. Mean percentage of normal, branched and truncated axons across three biological replicates (comprising n number of fish analyzed in total with 14 axons analyzed in each fish). Error bars represent the SD of the three replicates. Statistical analyses were done using one-way ANOVA test.

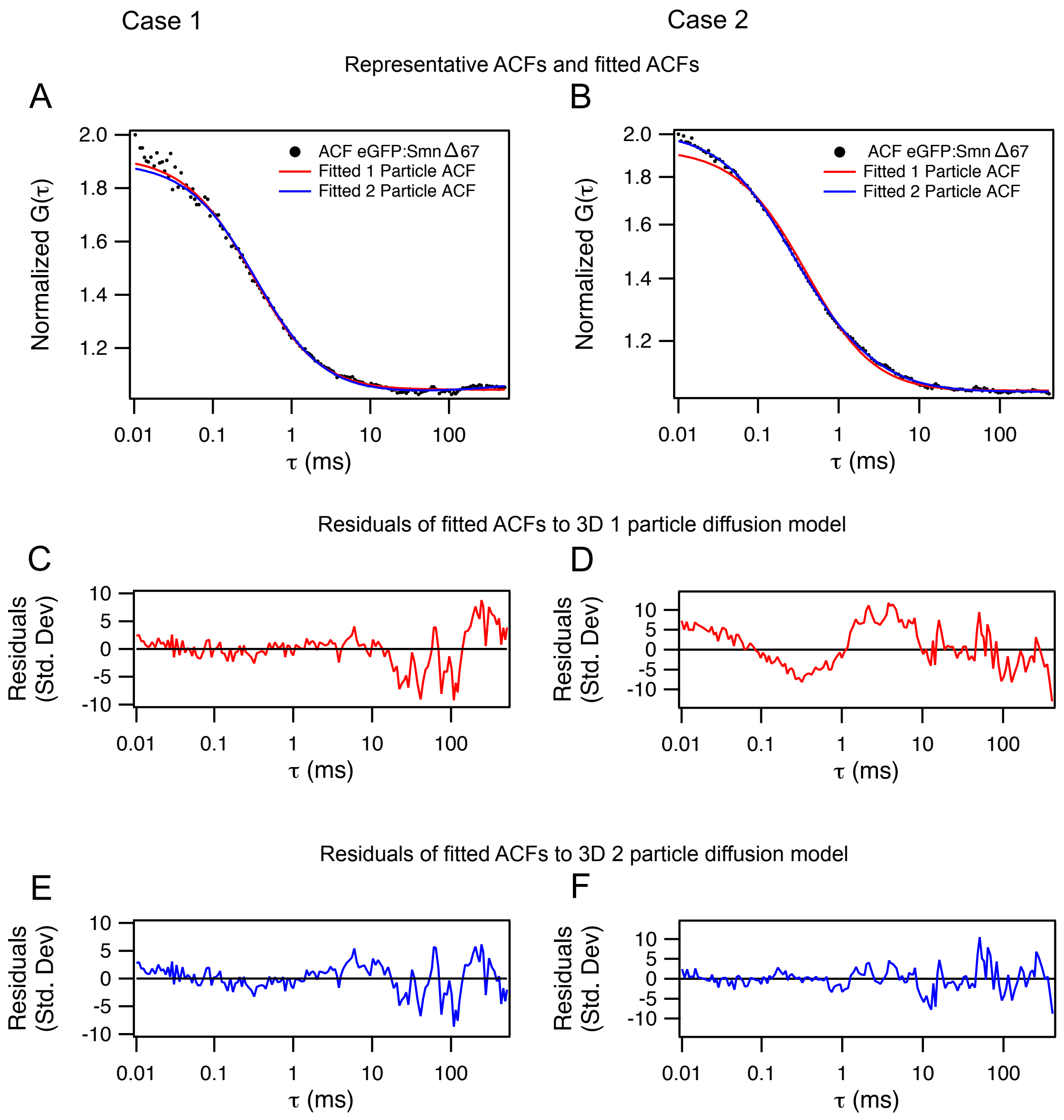

Figure S5. Comparison of the fitting models in Case 1 and Case 2

A, B. Representative ACFs for Case 1 and Case 2 fitted to both 3D 1-particle and 2-particle diffusion models. C, D. Residuals for a 3D 1-particle diffusion model fit. E, F. Residuals for a 3D 2-particle diffusion model fit. In Case 1, the fit quality shown by the residuals indicates that a 3D 1-particle fit was sufficient and a 2-particle fit did not improve the fitting quality considerably. Moreover, non-physical parameters were obtained for the 2-particle fit (negative fractions, non-consistent widely varying diffusion coefficients). In Case 2, a 2-particle diffusion model improved the fitting quality and thus was chosen.
