# Supplementary material for: Fluorescence Correlation Spectroscopy Reveals Survival Motor Neuron Oligomerization but No Active Transport in Motor Axons of a Zebrafish Model for Spinal Muscular Atrophy
Source: Front Cell Dev Biol. 2021 Aug 11;9:639904. doi: 10.3389/fcell.2021.639904 (PMC8385639; doi:10.3389/fcell.2021.639904)
Supplement: Supplementary Table 1 — Mean diffusion coefficients, D1, D2 and fraction of second components of mCherry, mCherry-Smn, mCherry-Smn: untagged Smn in cytoplasm and nucleus of SH-SY5Y cells. [file Table_1.docx]

Table S1. Mean diffusion coefficients, D_1_, D_2_ and fraction of second components of mCherry, mCherry-Smn, mCherry-Smn: untagged Smn in cytoplasm and nucleus of SH-SY5Y cells.

| **Sample** | **D_1_ ± SD (SEM)** **[μm^2^/s]** | **D_2_ ± SD (SEM)** **[μm^2^/s]** | **F_2_ ± SD** | **No. of Cells**  **(No. of pts)** |
| --- | --- | --- | --- | --- |
| **Measurements in cytoplasm** | | | | |
| mCherry  (cytoplasm) | 30.8 ± 15.3 (2.3) | - | - | 14 (46) |
| mCherry-Smn  (cytoplasm) | 18.7 ± 20.2 (3.6) | 0.91 ± 0.41 (0.07) | 0.35 ± 0.14 | 15 (31) |
| mCherry-Smn :  untagged Smn | 14.1 ± 16.8 (3.4) | 0.63 ± 0.43 (0.09) | 0.32 ± 0.11 | 9 (24) |
| **Measurements in nucleus** | | | | |
| mCherry | 33.4 ± 14.7 (2.9) | - | - | 11 (25) |
| mCherry-Smn | 19.2 ± 19.2 (4.2) | 0.35 ± 0.22 (0.05) | 0.35 ± 0.11 | 14 (22) |
| mCherry-Smn :  untagged Smn | 15.2 ± 16.8 (1.1) | 0.30 ± 0.19 (0.12) | 0.41 ± 0.13 | 8 (14) |
