# Supplementary material for: Fluorescence Correlation Spectroscopy Reveals Survival Motor Neuron Oligomerization but No Active Transport in Motor Axons of a Zebrafish Model for Spinal Muscular Atrophy
Source: Front Cell Dev Biol. 2021 Aug 11;9:639904. doi: 10.3389/fcell.2021.639904 (PMC8385639; doi:10.3389/fcell.2021.639904)
Supplement: Supplementary Table 2 — Diffusion coefficients, D1, D2 and fraction of second components, F2 for FCS measurements in cell body and axon. [file Table_2.docx]

Table S2. Diffusion coefficients, D_1_, D_2_ and fraction of second components, F_2_ for FCS measurements in cell body and axon.

| **Sample** | **D_1_ ± SD (SEM)** **[μm^2^/s]** | **D_2_ ± SD (SEM)** **[μm^2^/s]** | **F_2_ ± SD** | No. of Fish  (No. of pts) |
| --- | --- | --- | --- | --- |
| **Measurements in cell body** | | | | |
| eGFP | 27.0 ± 7.1 (1.3) | - | - | 9 (31) |
| eGFP-Smn | 28.4 ± 16.2 (4.3) | 0.41 ± 0.39 (0.11) | 0.60 ± 0.15 | 6 (14) |
| mCherry | 25.8 ± 12.5 (2.7) | - | - | 8 (22) |
| mCherry-Smn | 34.6 ± 21.6 (4.3) | 0.31 ± 0.12 (0.02) | 0.27 ± 0.14 | 11 (25) |
| **Measurements in axon** | | | | |
| eGFP | 26.8 ± 9.7 (2.4) | - | - | 6 (17) |
| eGFP-Smn | 26.0 ± 9.1 (2.6) | 0.35 ± 0.13 (0.04) | 0.72 ± 0.17 | 3 (12) |
| mCherry | 21.9 ± 10.7 (2.5) | - | - | 5 (19) |
| mCherry-Smn | 30.2 ± 20.0 (3.2) | 0.51 ± 0.22 (0.04) | 0.32 ± 0.11 | 12 (38) |
