# Supplementary material for: Fluorescence Correlation Spectroscopy Reveals Survival Motor Neuron Oligomerization but No Active Transport in Motor Axons of a Zebrafish Model for Spinal Muscular Atrophy
Source: Front Cell Dev Biol. 2021 Aug 11;9:639904. doi: 10.3389/fcell.2021.639904 (PMC8385639; doi:10.3389/fcell.2021.639904)
Supplement: Supplementary Table 3 — Diffusion coefficients, D1, D2 and fraction of second components, F2 for FCS measurements of mCherry-Smn under smn MO conditions. Data for uninjected control is taken from mCherry-Smn in Supplementary Table 2. [file Table_3.docx]

Table S3. Diffusion coefficients, D_1_, D_2_ and fraction of second components, F_2_ for FCS measurements of mCherry-Smn under *smn* MO conditions.

Data for uninjected control is taken from mCherry-Smn in Table S2.

| **Sample**  **(mCherry-Smn)** | **D_1_ ± SD (SEM)** **[μm^2^/s]** | **D_2_ ± SD (SEM)** **[μm^2^/s]** | **F_2_ ± SD** | No. of Fish  (No. of pts) |
| --- | --- | --- | --- | --- |
| **Measurements in cell body** | | | | |
| Uninjected Control | 34.6 ± 21.6 (4.3) | 0.31 ± 0.12 (0.02) | 0.27 ± 0.14 | 11 (25) |
| *smn* MO | - | 0.39 ± 0.24 (0.06) | - | 9 (16) |
| **Measurements in axon** | | | | |
| Uninjected Control | 30.2 ± 20.0 (3.2) | 0.51 ± 0.22 (0.04) | 0.32 ± 0.11 | 12 (38) |
| *smn* MO | 11.4 ± 10.9 (2.3) | 0.28 ± 0.14 (0.03) | 0.22 ± 0.11 | 14 (24) |
