# Supplementary material for: Fluorescence Correlation Spectroscopy Reveals Survival Motor Neuron Oligomerization but No Active Transport in Motor Axons of a Zebrafish Model for Spinal Muscular Atrophy
Source: Front Cell Dev Biol. 2021 Aug 11;9:639904. doi: 10.3389/fcell.2021.639904 (PMC8385639; doi:10.3389/fcell.2021.639904)
Supplement: Supplementary Table 4 — Diffusion coefficients of eGFP-SmnΔex6,7 compared to eGFP and eGFP-Smn in the cell body and axon. Data of eGFP and eGFP-Smn are taken from Supplementary Table 2. Measurements grouped under Case 1 were fitted to 3D 1-particle diffusion model, found in 6 out of 14 measurements, indicating a loss of the second component. Case 2 refers to measurements fitted to 3D 2-particle diffusion model, found in 8 out of 14 measurements. [file Table_4.docx]

Table S4. Diffusion coefficients of eGFP-SmnΔex6,7 compared to eGFP and eGFP-Smn in the cell body and axon.

Data of eGFP and eGFP-Smn are taken from Table S2.

Measurements grouped under Case 1 were fitted to 3D 1-particle diffusion model, found in 6 out of 14 measurements, indicating a loss of the second component. Case 2 refers to measurements fitted to 3D 2-particle diffusion model, found in 8 out of 14 measurements.

| **Sample**  **(Cell body)** | **D_1_ ± SD (SEM)** **[μm^2^/s]** | **D_2_ ± SD (SEM)** **[μm^2^/s]** | **F_2_ ± SD** | No. of Fish  (No. of pts) |
| --- | --- | --- | --- | --- |
| **Measurements in cell body** | | | | |
| eGFP | 27.0 ± 7.1 (1.3) | - | - | 9 (31) |
| eGFP-Smn | 28.4 ± 16.2 (4.3) | 0.41 ± 0.39 (0.11) | 0.60 ± 0.15 | 6 (14) |
| eGFP-SmnΔex6,7 | 18.5 ± 15.3 (5.1) | - | - | 5 (9) |
| **Measurements in axon** | | | | |
| eGFP | 26.8 ± 9.7 (2.4) | - | - | 6 (17) |
| eGFP-Smn | 26.0 ± 9.1 (2.6) | 0.35 ± 0.13 (0.04) | 0.72 ± 0.17 | 3 (12) |
| eGFP-SmnΔex6,7  Case 1: 43% | 15.3 ± 8.0 (3.2) | - |  | 5 (6) |
| eGFP-SmnΔex6,7  Case 2: 57% | 29.7 ± 11.5 (4.1) | 2.0 ± 1.4 (0.5) | 0.86 ± 0.11 | 3 (8) |
